# Neurocognitive performance in functional neurological disorder: A systematic review and meta‐analysis

**DOI:** 10.1111/ene.16386

**Published:** 2024-07-02

**Authors:** L. S. Merritt Millman, Isobel A. Williams, Johannes Jungilligens, Susannah Pick

**Affiliations:** ^1^ Institute of Psychiatry, Psychology and Neuroscience King's College London London UK; ^2^ Psychology in Healthcare, Newcastle Upon Tyne Hospitals NHS Foundation Trust and the Translational and Clinical Research Institute Newcastle University Callaghan UK; ^3^ Department of Neurology University Hospital Knappschaftskrankenhaus, Ruhr University Bochum Bochum Germany

**Keywords:** attention, functional motor, functional movement, neuropsycholog*, non*epileptic seizures

## Abstract

**Background and purpose:**

Cognitive complaints are common in functional neurological disorder (FND), but it is unclear whether objective neurocognitive deficits are present. This systematic review summarized validated/standardized cognitive test performance in FND samples across cognitive domains.

**Methods:**

Embase, PsycInfo and MEDLINE were searched from inception to 15 May 2023, combining terms for FND and cognitive domains (e.g., attention, memory, executive functioning). Studies included a range of FND phenotypes (seizures, motor, cognitive disorder, mixed), compared to healthy or clinical controls. Risk of bias was assessed with the modified Newcastle–Ottawa Scale and a qualitative synthesis/narrative review of cognitive performance in FND was conducted. Test performance scores were extracted, and random effects meta‐analyses were run where appropriate. This review was registered on PROSPERO, CRD42023423139.

**Results:**

Fifty‐six studies including 2260 individuals with FND were eligible. Although evidence for some impairments emerged across domains of executive functioning, attention, memory and psychomotor/processing speed, this was inconsistent across studies and FND phenotypes. Common confounds included group differences in demographics, medication and intellectual functioning. Only 24% of studies objectively assessed performance validity. Meta‐analyses revealed higher scores on tests of naming (*g* = 0.67, 95% confidence interval [CI] 0.50, 0.84) and long‐term memory (*g* = 0.43, 95% CI 0.13, 0.74) in functional seizures versus epilepsy, but no significant differences in working (*g* = −0.08, 95% CI −0.44, 0.29) or immediate (*g* = 0.25, 95% CI −0.02, 0.53) memory and cognitive flexibility (*g* = −0.01, 95% CI −0.29, 0.28).

**Conclusions:**

There is mixed evidence for objective cognitive deficits in FND. Future research should control for confounds, include tests of performance validity, and assess relationships between objective and subjective neurocognitive functioning.

## BACKGROUND

Functional neurological disorder (FND) encompasses neurological symptoms that are discernible from other disorders, both medical and neurological, on the basis of positive clinical signs [1]. Cognitive complaints are frequently reported in FND samples [2, 3] and have been associated with elevated psychopathology [2, 3], FND chronicity [4] and reduced health‐related quality of life [5].

In functional seizures (FS) [6, 7, 8], functional motor symptoms (FMS) [9, 10] and mixed FND samples [11, 12], objective difficulties across cognitive functions have been reported, including memory, executive functioning, attention/concentration, social–emotional cognition and language. Functional cognitive disorder (FCD), a specific subtype of FND involving enduring subjective cognitive difficulties paired with ‘internal inconsistency’ [13, 14], similarly has been associated with objective deficits in several cognitive domains [13, 15, 16, 17]. Further, some models of FND suggest that alterations in cognitive processing may play a mechanistic role in FND symptomatology [18, 19, 20].

Although there is some evidence for impaired objective cognitive performance in FND, these results are not always congruous [10, 16, 21, 22, 23, 24, 25, 26, 27], and few studies have explored both objective and subjective cognitive functioning in FND samples [3, 4, 27, 28]. This is especially relevant given a potential uncoupling between objective measures and subjective evaluations of cognitive performance in FND [3]. Mixed evidence for objective cognitive deficits presents difficulty for understanding the role of cognitive dysfunction in FND. A systematic review examining objective cognitive test performance in this population is needed.

### Aims

1. To summarize the available data on validated/standardized objective cognitive test performance in FND.
2. To gauge the cognitive performance profiles associated with FND subgroups.
3. To assess whether cognitive performance is related to clinical features (e.g., FND severity/chronicity, psychopathology).
4. To explore possible associations between objective and subjective cognitive outcomes in FND.
5. To evaluate the methodological quality of existing studies.

## METHODS

### Search strategy and selection criteria

This review was registered on PROSPERO (CRD42023423139) and follows Preferred Reporting Items for Systematic Reviews and Meta‐Analyses (PRISMA) guidelines [29]. Embase, PsycInfo and MEDLINE were searched from inception to 15 May 2023. Search terms are presented in Box 1. The reference lists of relevant reviews were manually searched and eligible studies published during the selection process were added.

**FIGURE 1:**
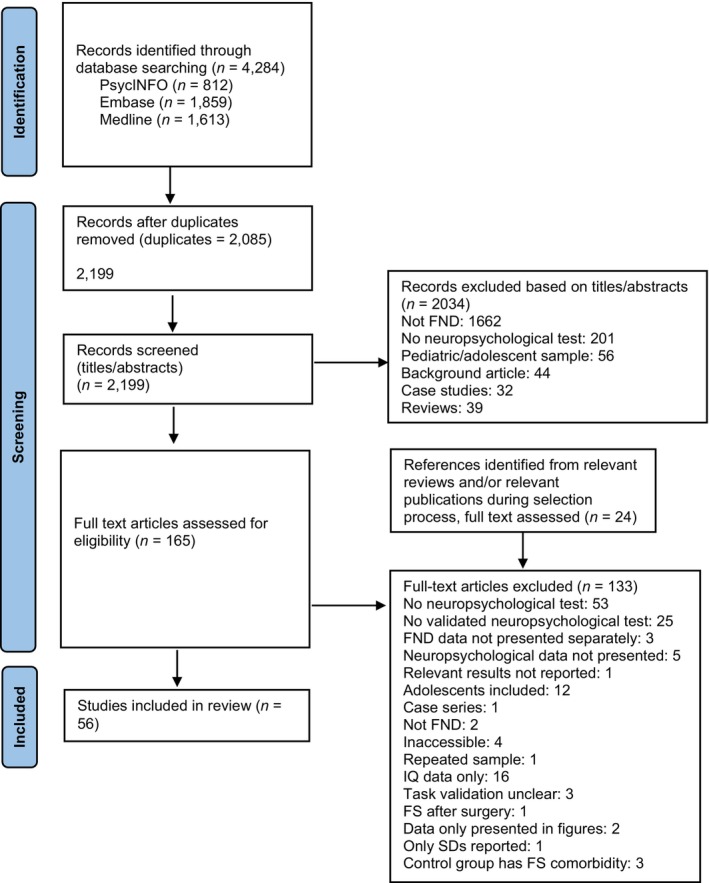
PRISMA flow diagram. FND, functional neurological disorder; FS, functional seizures; IQ, intelligence quotient; SD, standard deviation.

Inclusion and exclusion criteria are presented in Box 1. After removing duplicates, all titles and abstracts were screened by the first author, and those not relevant to the review were excluded. The remaining titles, abstracts and/or full texts were screened independently by two authors (LSMM, SP). Disparities were resolved by discussion with IAW and JJ. Reasons for exclusion are documented in Figure 1.

BOX 1 **Database search terms and inclusion/exclusion criteria**

*Searched in title*, *abstract and keyword fields across English and German studies*:

‘functional neurological’ OR ‘psychogenic’ OR ‘non*epileptic’ OR ‘conversion disorder’ OR ‘functional movement’ OR ‘functional cognitive’ OR ‘dissociative seizure’ OR ‘psychogenic non*epileptic seizure’ OR ‘functional motor’ OR ‘functional seizures’ OR ‘functional weakness’

AND ‘attention’ OR ‘memory’ OR ‘language’ OR ‘emotion*’ OR ‘social cognition’ OR ‘motor’ OR ‘neuropsych*’ OR ‘visual’ OR ‘spatial’ OR ‘executive’ OR ‘cognitive’ OR ‘IQ’ OR ‘flexibility’ OR ‘neuropsychological’ OR ‘neurocognitive’ AND ‘test*’ OR ‘scale’ OR ‘measure*’

*Inclusion*: adults (18+ years old) with confirmed FND diagnosis (via video electroencephalograph and/or by a consultant neurologist or neuropsychiatrist) of any type including FS, FMS, FCD, functional sensory symptoms and mixed FND symptoms; studies that used validated/standardized cognitive tests* covering any cognitive domain.

*Exclusion*: unpublished sources (pre‐prints, theses), conference abstracts, studies not including an already validated/standardized cognitive test, studies only including performance validity tests (PVTs) or self‐report questionnaires, studies of mixed samples (FND participants alongside psychiatric/physical health conditions), reviews and/or meta‐analyses.

*Cognitive tests were included if they had been previously validated in healthy, neurological and/or psychiatric populations, displaying sound psychometric properties (see Supplementary References and Table S3 for all included tests and validation studies). Any studies using amended, updated or altered versions of previously validated/standardized tasks, or novel/unvalidated cognitive tests, were not eligible for inclusion.

The following data were extracted for each study: authors, year, geographical location, FND phenotype, control sample, age, gender ratio, cognitive test/s, cognitive domain/s, raw and/or standardized cognitive scores and between‐group comparisons (Tables S3 and S4). When data (mean/standard deviation) for cognitive tests were not reported, authors were contacted with a request to provide this. Correlations between clinical variables, cognitive test scores and subjective cognitive symptoms, and data regarding possible confounding variables, were also extracted.

Included studies were reviewed for quality and risk of bias by two independent raters (LSMM and IAW/JJ/SP) using modified Newcastle–Ottawa Scales [30, 31] (Table S1). Disagreements between raters were resolved by group discussion.

### Data analysis

Data from eligible studies were synthesized narratively, organized by cognitive domain and FND phenotype. Definitions of superior, normal, deficient or impaired performance imposed by the study authors were adhered to, resulting in some definitions varying from study to study. Random effects meta‐analyses were conducted in R [32] across studies using the same cognitive test in the same FND phenotype (≥five studies per test, per group). All studies were required to provide mean/standard deviation for at least one FND and one control group. Hedges’ *g* effect size and 95% confidence intervals were calculated for each study, with forest plots for visualization of the data and funnel plots and Egger's test [33] to assess publication bias. Heterogeneity was assessed using the *I* ^2^ statistic [34]. The funder of the study had no role in study design, data collection, data analysis, data interpretation or writing of the report.

## RESULTS

Fifty‐six studies including 2260 individuals with FND were eligible (Table S3; Figure 1). Thirty‐two studies included FS samples (*n* = 1525), 10 with FMS (*n* = 267), nine with FCD (*n* = 368) and five with mixed FND (*n* = 131). Most were case–control designs (*k* = 51), including healthy controls (HCs) (*k* = 14), neurological controls (NCs) (*k* = 22) or both (*k* = 15). The cognitive domains assessed were general cognitive functioning (*k* = 20), executive functioning (*k* = 26), attention (*k* = 20), memory (*k* = 29), language (*k* = 16), psychomotor/processing speed (*k* = 13), visuospatial abilities (*k* = 12) and social cognition (*k* = 8). Twenty studies reported raw scores, 10 reported standardized scores, five reported a mix, and 21 were unclear regarding the scores presented.

Thirty‐six studies were rated as low risk of bias, 17 as high risk of bias and three as very high (Table S1). Biases included non‐consecutive/selective recruitment, insufficient control of confounding variables (e.g., age, medication, education, intelligence), previous head injury/trauma, uncertain ascertainment of diagnosis, lack of standardization of test scores/comparison of scores to normative data.

### Global cognitive functioning

In FS, one study found below average global cognitive performance (as measured with cognitive screening tools) compared to age‐matched norms [35], and another reported impaired‐abnormal functioning (scores >1–1.5SD from standardized mean) in 18.8%–49.1% of FS participants with and without ictal motor symptoms [36]. Individuals with FS performed similarly to NCs across five studies [37, 38, 39, 40, 41] although two other studies found significantly higher levels of global cognitive functioning in FS versus NCs [21, 42]. Three studies of FMS either specifically matched groups (FMS and HCs) for global cognitive functioning [25] or ensured that all participants scored above the cut‐off [43, 44], although the FMS group scored significantly lower than HCs in one study [44]. Another study found lower scores in FMS versus HCs [45], but no difference between FMS and NCs [45]. FCD samples displayed significantly lower global cognitive functioning than HCs in one study [17], but performed no differently to HCs [46] or NCs [17, 27, 47] across four others, although scores were below the cut‐offs in some cases [13, 17, 27, 48]. In contrast, another study found significantly better functioning in FCD compared to NCs (neurodegenerative disorders like dementia), but not in comparison to those with mild cognitive impairment [48], while O'Malley et al. [46] revealed better functioning in FCD compared to both individuals with Alzheimer's disease and individuals with mild cognitive impairment.

### Psychomotor and processing speed

Compared to HCs, FS samples exhibited slower motor speed [6, 49]. Although no differences were seen between FS and NCs [8, 24, 42], FS scores were in the below average range in one study [8]. In FMS compared to HCs, two studies found no differences regarding processing speed or reaction times [9, 10], but two others reported slow processing/motor speed in FMS versus HCs [44, 50] and NCs [50]. In FCD, one study found that all individuals performed within the normal range on a test of information processing speed [51], whereas another reported inferior performance (<25th percentile) in approximately half of their FCD sample [16]. One study of mixed FND found no difference from HCs for sensorimotor and information processing speed [3], but a second reported deficit/disorders (<20th percentile) of information processing and psychomotor speed in 55.5%–88.5% of their mixed FND sample compared to normative data, with performance significantly worse than individuals with other somatic symptoms and related disorders [12].

### Attention

Poorer sustained, visual and/or auditory attention was seen in FS versus HCs [7, 52, 53], with overall scores significantly below norms [35] or in the below average range compared to age‐matched normative data [8] in FS, although one study reported no difference between these groups [54]. Across four studies, weaker attention was seen in FS versus NCs [52, 53, 55], specifically auditory and spatial [8], although other studies reported no differences in visual attention between groups [36, 37, 39]. In FMS, one study found no overall difference in visual attention between FMS, HCs and NCs [9] and a second study found worse visual attention in FMS versus HCs [44]. A third study found poorer performance in FMS compared to HCs and NCs, with performance suggesting a failure in the inhibition of distractor information [56]. In some mixed FND samples, decreased focused, sustained and/or visual attention was seen compared to HCs [11, 57, 58] and reduced sustained attention was seen compared to psychiatric controls [11]. Deficits (<2.4th percentile) across tests of divided, selective and sustained attention were present in 12%–36% of another mixed FND sample [12], with performance significantly worse than those with other somatic symptoms and related disorders on a test of selective attention. In contrast, Pick et al. [3] found no difference between FND and HCs in sustained attention.

### Memory

Individuals with FS exhibited poorer performance on tests of verbal learning and memory compared to HCs [7, 59], with another study reporting scores below age‐matched norms on tests of immediate memory and delayed recall [35]. In contrast, one study reported no difference in short‐term memory between FS and HCs [22] and another found better memory for complex visual scenes in FS versus HCs [60]. Compared to NCs, better performance on tests of verbal, visuospatial, delayed recall and/or recognition was seen in FS [8, 24, 36, 42, 61, 62], although one study reported below average scores in FS [8]. However, seven studies found no differences between FS and NCs on tests of verbal learning and logical memory [6, 37, 39, 59, 63, 64, 65]. Myers et al. [66] reported poorer delayed verbal memory for stories (logical memory) in FS + PTSD compared to FS without trauma or FS + trauma without PTSD, and Bortz et al. [67] found variable performance in FS versus NCs. Three studies of FMS found no differences from HCs across tests of visual and verbal memory [9, 10, 44], although FMS performance was significantly below the expected level on a subtest of immediate reproduction/verbal memory capacity [9, 44]. ‘Inferior’ (<25th percentile) performance was seen in approximately half of an FCD sample on a test of visual and verbal memory [16]. Compared to HCs, FCD participants performed significantly worse on a test of verbal learning and memory but exhibited superior performance on delayed recall and retention versus NCs [15]. In contrast, another study reported that all individuals with FCD scored within the range defined as non‐impaired performance on a test of word list learning [51]. In three studies, mixed FND groups performed significantly worse than HCs [11, 57, 58] and psychiatric controls [11] across tests of learning and logical memory. Compared to normative data, de Vroege et al. [12] found that 23.1%–53.9% of those with mixed FND had deficits/disorders (<20th percentile) on tests of verbal and visual memory.

### Visuospatial

There were no significant differences in performance between FS, HCs [39] and NCs [24, 39, 63, 64, 65] on tests of copying or spatial visualization. Krámská et al. [35] reported scores below the norms in FS; however, Pick et al. [60] found significantly better performance in FS compared to HCs on a test of general object perception/recognition. Two studies in FMS reported no differences in visuospatial perception and copying complex figures between FMS and HCs [10, 44] and, in one study of FCD, Bhome et al. [16] found that 86% of their sample displayed intact visuospatial perception performance (25th–75th percentiles). In mixed FND, performance was significantly poorer compared to HCs, psychiatric controls [11] and other somatic symptoms and related disorders [12] on tests of reproduction and visuospatial perception and, compared to normative scores, deficits/disorders (<20th percentile) were present in 100% of another mixed FND sample [12].

### Executive functioning

One study of FS reported similar performance on a test of inhibition between FS and HCs [23], although five others found poorer executive functioning (inhibitory control, working memory, flexibility) in FS versus HCs [6, 7, 52, 53, 54]. Eight studies reported no significant differences between FS and NCs [6, 24, 37, 39, 62, 63, 64, 65] (working memory, set‐shifting/flexibility, fluency), in contrast to three others revealing reduced inhibitory control and mental flexibility compared to NCs [8, 52, 53]; however, another study reported superior mental flexibility in FS versus one NC group [42]. There were no differences in set‐shifting and inhibition between FS samples without trauma compared to FS + trauma and FS + PTSD subgroups [66]. Although one study found significantly worse performance in FMS versus HCs (working memory, fluency, flexibility) [44], two others revealed no difference between these groups (flexibility, inhibition, fluency) [9, 10]. However, Voon et al. [10] reported elevated commission errors in motor response inhibition in FMS versus HCs. Individuals with FCD exhibited poorer mental flexibility compared to HCs but performed no differently to NCs [15]. Significantly worse performance (flexibility, inhibition) was seen in mixed FND versus HCs [11, 57, 58] and psychiatric controls [11]. However, another study reported no significant differences on tests of working memory, set‐shifting and cognitive flexibility between mixed FND and HCs [3], and a fifth reported deficits/disorders (<20th percentile) in 7.7%–22.2% of their sample [12] and significantly worse performance on a test of phonological verbal fluency compared to those with other somatic symptoms and related disorders.

### Language

Superior performance on tests of confrontational naming was seen in FS versus NCs [8, 24, 36, 62] alongside higher scores on a test of single‐word reading [48], with no differences in confrontational naming [6, 37, 39, 63, 65] or reading [37, 46] in five other studies, and no differences in confrontational naming among FS samples without trauma, with trauma and with PTSD [66]. There were similarly no differences between FS and NCs across tests of semantic verbal fluency [56, 58], receptive language [48] and verbal comprehension [37], although Krámská et al. [35] reported scores below the norms in FS on tests of picture naming and semantic fluency. Two studies exploring confrontational naming in FMS reported similar scores among FMS, HCs [10, 26] and NCs [26]. In contrast, a third study reported significantly worse performance in FMS versus HCs (confrontational naming, semantic verbal fluency) [44]. In mixed FND, performance on a confrontational naming test was no different to other somatic symptoms and related disorders, although 40% of the FND group displayed deficits/disorders, compared to normative reference data (<20th percentile) [12].

### Social cognition

One study reported poorer performance in FS versus HCs [68], in contrast to three studies revealing no difference in social cognition between FS and HCs [22, 23, 69] (processing of facial stimuli, emotion recognition). Similarly, there was no difference in emotion recognition between FS and NCs in one study [68], but significantly poorer affective perception/expression in FS in another [62]. In FMS, although one study reported poorer emotion recognition in FMS versus HCs [43], a second study found no significant difference compared to both HCs and NCs [70]. A mixed FND group was no different to HCs in the recognition of facial emotions but exhibited shorter reaction times compared to HCs on an anger version of an emotional bias task [3].

### Possible influence of confounding variables

A proportion of included studies did not match groups for age, and did not account for age in the analyses of cognitive test scores. Furthermore, some studies did not consider educational background or match groups for years of education. Historical head/brain injury or abnormal structural neuroimaging findings were present in some FND samples, with some of these samples displaying inferior test performance compared to HCs/normative data.

Many studies included participants taking medications with potential cognitive implications (e.g., antiseizure medication, benzodiazepines, opioids), and some did not report on medication. Notably, eight studies with FND samples taking medications potentially affecting cognition revealed poorer performance on cognitive tests compared to HCs or normative data.

Twenty‐one studies included a validated measure of intelligence (Tables S3 and S4), with only six mentioning intelligence quotient (IQ) scores <70 as an exclusion criterion. In some reports, IQ was correlated positively with performance on tests of executive functioning, memory and attention, and when estimated IQ was added as a covariate, many significant results across cognitive tests disappeared.

Some investigators measured performance validity (see Table S2 for tests used and scores across groups) to establish whether assessment results were likely to accurately reflect true cognitive abilities [71]. Two studies reported significantly more failed/invalid performance in FS versus NCs [38, 72], and some found that individuals displaying worse or invalid PVT performance exhibited greater impairment across cognitive tests [35, 38, 72]. Significant relationships were present between performance validity outcomes and education [35], immediate recall and depression [9], emotional functioning [61], learning and memory [6] and greater quality‐of‐life complaints and neurological symptoms [72].

### Correlations between objective and subjective cognitive performance and clinical variables

Prigatano and Kirlin [62] found a significant correlation between language scores and subjective judgements of word‐finding ability in NCs, but not in FS. In FMS, subjective cognitive complaints were not related to objective cognitive performance [44], but social cognition was directly correlated with verbal fluency scores in another study (FMS and HCs) [43]. Pick et al. [3] reported a significant correlation between objective set‐shifting performance and subjective performance ratings in HCs, but this relationship was not significant in mixed FND. Correlates of self‐reported memory or attention disturbances and cognitive complaints included depression [3, 40, 44, 58, 62], anxiety [44, 58, 62], somatoform and psychological dissociation [3], age [27], seizure frequency [40] and performance ratings on sustained attention and working memory tests [3].

Objective test performance in one or more domains was significantly negatively associated with depression, anxiety and disease duration in some samples. Alexithymia scores were positively correlated with spatial working memory in FS [7] and, when childhood trauma was added as a covariate, the difference between FS and HCs on a test of executive functioning was no longer significant [54]. In FCD, 40% of individuals with depressive symptoms displayed invalid patterns of results across neuropsychological tests [13].

### Meta‐analysis

**FIGURE 2:**
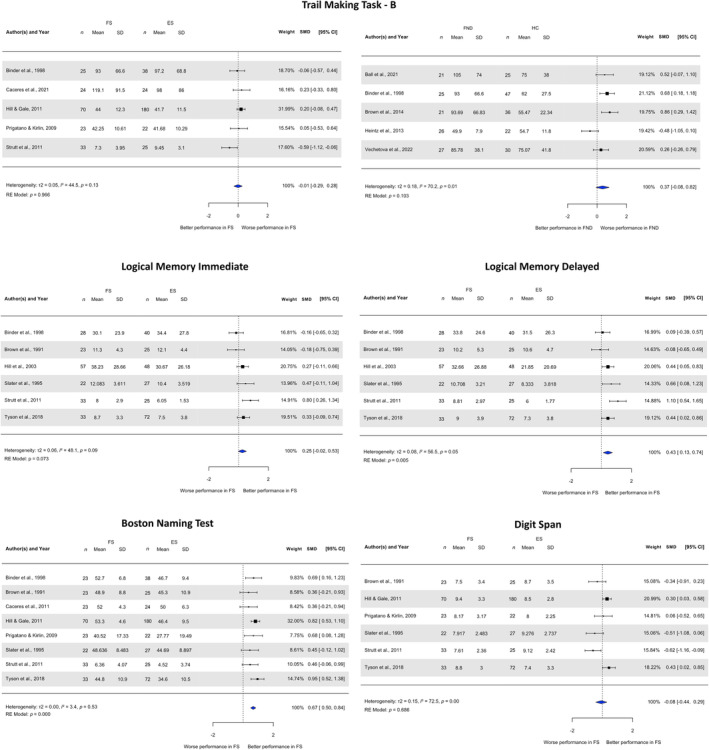
Meta‐analyses of executive functioning, memory, naming and working memory in FS versus ES and FND versus HCs. ES, epilepsy; FND, functional neurological disorder; FS, functional seizures; HCs, healthy controls; RE, random effects; SD, standard deviation; SMD, standardized mean difference.

Random effects meta‐analysis exploring executive functioning (cognitive flexibility; Trail Making Task B, TMT‐B [73]) in FS versus epilepsy (ES) (*k* = 5, *n* = 464; Figure 2) showed no difference between groups. Moderate heterogeneity was present, with no outliers and no evidence of publication bias (Egger's test: *z* = −0.69, *p* = 0.49; Figure S1). The included studies were all rated as low risk of bias. In mixed FND samples (FS, FMS, FCD, mixed FND) versus HCs (*k* = 5, *n* = 280), there was again no consistent difference between groups in TMT‐B performance. Heterogeneity was high, with no outliers and no evidence of publication bias (Egger's test: *z* = −0.57, *p* = 0.57). Three studies in this analysis were low risk of bias and two were high risk of bias, with a meta‐regression revealing no significant effect of risk‐of‐bias category (*p* = 0.55).

A random effects meta‐analysis examining confrontational naming ability (Boston Naming Test [74]) in FS versus ES (*k* = 8; *n* = 663; Figure 2) found superior performance in FS. There was low heterogeneity, no outliers, no evidence of publication bias (Egger's test: *z* = −1.76, *p* = 0.08), and all studies were rated as low risk of bias.

Random effects meta‐analysis of six studies (*n* = 555) including the Digit Span subtest [75] revealed no difference in sustained attention and working memory between ES and FS (Figure 2). High heterogeneity was present, alongside possible publication bias (Egger's test: *z* = −2.04, *p* = 0.04), but there were no outliers in the analysis and all included studies were rated as low risk of bias.

Two random effects meta‐analyses examined immediate (*k* = 6, *n* = 433) and delayed (*k* = 6, *n* = 433; Figure 2) logical memory (recall of stories; Wechsler Memory Scale [76]) in FS versus ES. There was no clear difference between groups in immediate memory; however, those with FS significantly outperformed those with ES on delayed memory, exhibiting higher scores across five studies. Moderate heterogeneity was present, there were no outliers and there was no evidence of publication bias (Immediate memory Egger's test, *z* = 0.06, *p* = 0.95; Delayed memory Egger's test, *z* = 0.29, *p* = 0.77) in both meta‐analyses. All included studies were rated as low risk of bias.

## DISCUSSION

There is inconclusive evidence and significant heterogeneity regarding objective cognitive deficits in FND; however, meta‐analyses of scores on confrontational naming and delayed verbal recall tests indicate superior performance in FS compared to ES. Very few studies explored associations between objective and subjective cognitive outcomes in FND, and the methodological quality of existing studies is variable.

In FS and FMS, there was inconsistent evidence regarding objective deficits in global cognitive functioning, executive functioning, language, psychomotor/processing speed and social cognition. Specific impairments in inhibitory control and mental flexibility, sustained, visual and/or auditory attention, and verbal learning and memory were seen in some FS studies. Meta‐analyses revealed superior performance on tests of language and long‐term memory in FS compared to ES, but no clear signal and high heterogeneity regarding attention, working or immediate memory and cognitive flexibility. Superior long‐term memory and naming in FS may be expected given the possibility of seizure‐related neurological damage in ES [77]. In FMS, deficits in attention included a failure to inhibit distractor information, memory impairments centred on immediate reproduction/verbal memory capacity and one study that suggested a specific deficit in motor response inhibition. In FCD, there was mixed evidence for inferior psychomotor/processing speed, memory, general cognitive functioning and executive functioning compared to HCs and NCs. Impairments in memory, visuospatial perception, attention (focused/sustained/visual) and executive functioning (flexibility, inhibition) were seen in some mixed FND samples. Although heterogeneous results were seen across the studies included in this review, there is some alignment with models of FND proposing a mechanistic role for alterations in cognitive processing, specifically reduced mental flexibility or inhibitory control, and disruptions in attention [3, 18, 19, 20].

Objective and subjective cognitive measures were not correlated in FND across three studies [3, 44, 62], and the presence of elevated subjective cognitive symptoms/complaints paired with relatively normal objective functioning [3] suggests a disconnect between self‐rated and objective performance in FND. This discrepancy may be of particular importance in FND compared to other neurological or medical disorders, for example. Metacognitive alterations may be present [4, 78, 79, 80], which can be assessed by consistently measuring both objective and subjective functioning in future research; this would generate a better understanding of the potential role of metacognition in FND, both local (moment‐to‐moment estimates of task performance) and global (longer‐term estimates of overall performance). Further, associations between self‐reported cognitive disturbances and psychopathology (i.e., depression, anxiety, dissociation), as well as FND severity, highlight potentially important relationships between these variables which require further exploration.

Methodological inconsistencies were present, with 19 studies finding significant differences on cognitive tests that may have been explainable by confounding variables. In particular, 62% of studies included participants taking medications with potential cognitive implications, and 27% did not mention medication at all. Similarly, historical head/brain injury or abnormal structural neuroimaging findings were also not considered in 13% of included studies. Mild brain injuries are common in some FND phenotypes (e.g., FS [2, 81]) and, given the cognitive and psychological outcomes associated with traumatic brain injury [82], may have influenced the results presented in some of the included studies. These potential confounds are significant limitations in this literature and warrant careful consideration in future studies. Such methodological inconsistencies may have played a significant role in the heterogeneity of results seen within this review.

There is also the possibility of selective reporting of findings or lack of publication of results revealing no differences between FND and control groups within the literature more broadly. Equally, it is important to note that neurological controls encompassed a range of disorders (Table S3), which may affect the interpretation of cognitive test performance in FND. Careful consideration should be given when selecting the most appropriate control groups in relation to study aims and cognitive tests being administered. Further, some common cognitive tests assess a range of domains for which they may not have been explicitly validated. Future selection of tests should be based on their validation for assessing specific cognitive domains.

Performance validity tests were only included in 24% of studies and there was considerable variability in results across studies, with one study showing that PVTs embedded within cognitive tests were more effective at differentiating between groups, compared to free‐standing PVTs [24]. Associations between performance validity and education, psychopathology, quality of life, and certain aspects of cognition reveal the importance of measuring task engagement alongside cognitive tests [71]. Data from individuals failing PVTs should be excluded or accounted for in analyses of cognitive test scores. Inclusion of both embedded and free‐standing PVTs would be valuable in future cognitive research in FND.

Despite the subjective cognitive complaints commonly present in FND, objective impairment in cognitive performance is inconsistent across FND samples. Future research should aim towards careful control of confounds, the inclusion of PVTs, and assessment of both subjective and objective cognitive functioning in specific FND phenotypes.

## AUTHOR CONTRIBUTIONS

**L. S. Merritt Millman:** Investigation; writing – original draft; methodology; validation; visualization; writing – review and editing; formal analysis; data curation. **Isobel A. Williams:** Methodology; validation; writing – review and editing. **Johannes Jungilligens:** Methodology; validation; writing – review and editing. **Susannah Pick:** Conceptualization; funding acquisition; writing – original draft; methodology; validation; writing – review and editing; formal analysis; supervision; resources.

## FUNDING INFORMATION

SP and LSMM were funded by a Medical Research Council Career Development Award to SP [MR/V032771/1].

## CONFLICT OF INTEREST STATEMENT

None.

## Supporting information


Data S1.

## Data Availability

The data that support the findings of this study are available from the corresponding author upon reasonable request.
