# Supplementary material for: Neurocognitive performance in functional neurological disorder: A systematic review and meta‐analysis
Source: Eur J Neurol. 2024 Jul 2;32(1):e16386. doi: 10.1111/ene.16386 (PMC11618116; doi:10.1111/ene.16386)
Supplement: Supplementary file 1 — Data S1. [file ENE-32-e16386-s001.docx]

**Supplementary Table 1. Newcastle Ottawa Scale Risk of Bias Ratings**

| **Case-control Studies** | | | | | | | | | | |
| --- | --- | --- | --- | --- | --- | --- | --- | --- | --- | --- |
| Study | **Selection** | | | | **Comparability** | | **Exposure** | | | **Total Score (0-9)** |
|  | Is the case definition adequate?    a) yes, with independent validation*  b) yes, e.g. record linkage or based on self-reports  c) no description | Representativeness of the cases  a) consecutive or obviously representative series of cases*  b) potential for selection biases or not stated | Selection of Controls  a) community controls*  b) hospital controls*  c) no description | Definition of Controls  a) no history of FND (endpoint)*  b) no description of source | Comparability of cases and controls on the basis of the design or analysis  a) study controls for age (select the most important factor)* | Comparability of cases and controls on the basis of the design or analysis (Other factors)  b) study controls for any additional factor (this criteria could be modified to indicate specific control for a second important factor)* | Ascertainment of exposure  a) secure record (e.g., surgical records) vEEG*  b) consultant neurologist or neuropsychiatrist*  c) self-report (or mixture)  d) no description | Same method of ascertainment for cases and controls  a) yes*  Patient controls: Consultant or neuropsychiatrist confirmed dx/ vEEG  b) no/self-report | Non-Response rate  a) same rate for both groups*  b) missing data reported and dealt with appropriately*  c) missing data not reported or accounted for |  |
| Almis et al., 2013 | 1 | 0 | 1 | 0 | 1 | 1 | 1 | N/A | 1 | 6/8 |
| Ball et al., 2021 | 1 | 1 | 1 | 1 | 1 | 1 | 1 | 1 | 0 | 8/9 |
| Bharambe & Larner, 2018 | 1 | 1 | 1 | 1 | 0 | 1 | 1 | 1 | 0 | 7/9 |
| Binder et al., 1998 | 1 | 1 | 1 | 1 | 1 | 1 | 1 | 1 | 0 | 8/9 |
| Borelli et al., 2022 | 0 | 1 | 1 | 0 | 0 | 1 | 1 | 1 | 0 | 5/9 |
| Bortz et al., 1995 | 1 | 0 | 1 | 1 | 1 | 1 | 1 | 1 | 0 | 7/9 |
| Brown et al., 1991 | 1 | 1 | 1 | 1 | 1 | 1 | 1 | 1 | 0 | 8/9 |
| Brown et al., 2014 | 1 | 0 | 1 | 1 | 1 | 1 | 1 | N/A | 0 | 6/8 |
| Caceres et al., 2021 | 1 | 1 | 1 | 1 | 1 | 0 | 1 | 1 | 1 | 8/9 |
| Criswell et al., 2010 | 1 | 1 | 1 | 1 | 1 | 0 | 1 | 1 | 0 | 7/9 |
| De Vroege et al., 2021 | 1 | 1 | 1 | 1 | 1 | 1 | 1 | 1 | 0 | 8/9 |
| Demartini et al., 2014 | 1 | 0 | 1 | 1 | 1 | 1 | 1 | 1 | 0 | 7/8 |
| Demartini et al., 2019 | 1 | 0 | 1 | 0 | 1 | 1 | 1 | N/A | 0 | 5/8 |
| Demir et al., 2013 | 1 | 1 | 1 | 1 | 1 | 1 | 1 | 1 | 0 | 8/9 |
| Drane et al., 2006 | 1 | 1 | 1 | 1 | 1 | 1 | 1 | 1 | 0 | 8/9 |
| Dunbar et al., 2021 | 1 | 1 | 1 | 1 | 1 | 1 | 1 | 1 | 1 | 9/9 |
| Giugno et al., 2023 | 1 | 1 | 1 | 1 | 1 | 0 | 1 | 1 | 0 | 7/9 |
| Hamouda et al., 2021 | 1 | 0 | 1 | 0 | 1 | 1 | 1 | N/A | 1 | 6/8 |
| Gursoy et al., 2021 | 1 | 0 | 1 | 1 | 1 | 1 | 1 | 0 | 1 | 7/9 |
| Heintz et al., 2013 | 1 | 0 | 1 | 1 | 1 | 1 | 1 | 1 | 0 | 7/9 |
| Hill et al., 2003 | 1 | 1 | 1 | 1 | 1 | 1 | 1 | 1 | 0 | 8/9 |
| Hill & Gale, 2011 | 1 | 0 | 1 | 1 | 1 | 1 | 1 | 1 | 1 | 8/9 |
| Huys et al., 2020 | 1 | 0 | 1 | 1 | 1 | 1 | 1 | 1 | 1 | 8/9 |
| Irorutola et al., 2020 | 1 | 0 | 1 | 1 | 1 | 1 | 1 | N/A | 0 | 6/8 |
| Leon-Sarmiento et al., 2019 | 1 | 1 | 1 | 1 | 1 | 1 | 1 | 1 | 0 | 8/9 |
| Lloyd et al., 2022 | 1 | 1 | 1 | 1 | 0 | 0 | 1 | 1 | 1 | 7/9 |
| Matthews et al., 2020 | 1 | 0 | 1 | 1 | 1 | 1 | 1 | 1 | 0 | 7/9 |
| McWhirter et al., 2021 | 1 | 1 | 1 | 1 | 0 | 1 | 0 | 0 | 0 | 5/9 |
| O’Brien et al., 2015 | 1 | 0 | 1 | 1 | 1 | 1 | 1 | N/A | 1 | 7/8 |
| O’Malley et al., 2020 | 1 | 0 | 1 | 0 | 0 | 0 | 1 | 0 | 0 | 3/9 |
| Ozer Celik et al., 2015 | 1 | 0 | 1 | 0 | 1 | 1 | 1 | 1 | 1 | 7/9 |
| Pennington et al., 2019 | 1 | 0 | 1 | 1 | 0 | 1 | 1 | 1 | 0 | 6/9 |
| Pick, Mellers, & Goldstein, 2016 | 1 | 1 | 1 | 1 | 1 | 1 | 1 | N/A | 1 | 8/8 |
| Pick, Mellers, & Goldstein, 2018a | 1 | 0 | 1 | 1 | 1 | 1 | 1 | N/A | 1 | 7/8 |
| Pick, Mellers, & Goldstein, 2018b | 1 | 0 | 1 | 1 | 1 | 1 | 1 | N/A | 0 | 6/8 |
| Pick et al., 2023 | 1 | 1 | 1 | 1 | 1 | 1 | 1 | N/A | 1 | 8/8 |
| Prigatano & Kirlin, 2009 | 1 | 0 | 1 | 1 | 1 | 1 | 1 | 1 | 0 | 7/9 |
| Sackellares & Sackellares, 2001 | 1 | 0 | 1 | 1 | 0 | 1 | 1 | N/A | 0 | 4/8 |
| Salinsky et al., 2020 | 1 | 1 | 1 | 1 | 0 | 0 | 1 | 1 | 1 | 7/9 |
| Silveri et al., 2022 | 1 | 1 | 1 | 1 | 1 | 1 | 1 | N/A | 0 | 7/8 |
| Simani et al., 2020a | 1 | 0 | 1 | 1 | 1 | 0 | 1 | 1 | 0 | 6/9 |
| Simani et al., 2020b | 1 | 0 | 1 | 1 | 1 | 0 | 1 | 1 | 0 | 6/9 |
| Slater et al., 1995 | 1 | 0 | 1 | 1 | 1 | 1 | 1 | 1 | 1 | 8/9 |
| Strutt et al., 2011 | 1 | 1 | 1 | 1 | 1 | 1 | 1 | 1 | 0 | 8/9 |
| Tyson et al., 2018 | 1 | 0 | 1 | 1 | 1 | 1 | 1 | 1 | 0 | 7/9 |
| Vechetova et al., 2022 | 1 | 0 | 1 | 1 | 1 | 1 | 1 | N/A | 0 | 6/8 |
| Voon et al., 2013 | 1 | 0 | 1 | 1 | 1 | 1 | 1 | N/A | 0 | 6/8 |
| Walterfang et al., 2011 | 1 | 1 | 1 | 1 | 0 | 0 | 1 | 1 | 1 | 7/9 |
| Wilkus & Dodrill, 1989 | 1 | 1 | 1 | 1 | 1 | 1 | 1 | 1 | 0 | 8/9 |
| Wilkus, Dodrill, & Thompson, 1984 | 1 | 0 | 1 | 1 | 0 | 0 | 1 | 1 | 0 | 5/9 |
| Ye et al., 2020 | 1 | 1 | 1 | 1 | 0 | 0 | 1 | 1 | 1 | 7/9 |
| **Cross-sectional Studies** | | | | | | | | | | |
|  | **Selection** | **Comparability** | **Outcome** | **Total Score (0-8)** |  |  |  |  |  |  |
|  | Representativeness of the sample  a) truly representative of the average in the target population* (all subjects or random sampling)  b) somewhat representative of the average in the target population* (non-random sampling)  c) selected group of users  d) no description of the sampling strategy | Sample Size  a) justified and satisfactory*  b) not justified | Ascertainment of exposure  a) validated measurement tool*  b) non-validated measurement tool, but the tool is available or described*  c) no description of the measurement tool | Non-respondents  a) comparability between respondents and non-respondents characteristics is established, and the response rate is satisfactory*  b) the response rate is unsatisfactory, or the comparability between respondents and non-respondents is unsatisfactory  c) no description of the response rate or the characteristics of the responders and non-responders | Comparability  a) the study controls for the most important factor (select one)* | Comparability (other factors)  b) the study controls for any additional factor* | Assessment of outcome  a) standardized outcome measure (standardized test)*    d) self-report    e) no description | Statistical test  a) The statistical test used to analyse the data is clearly described and appropriate, and the measurement of the association is presented, including confidence intervals and the probability level (p value)*  b) The statistical test is not appropriate, not described or incomplete |  |  |
| Bhome et al., 2019 | 1 | 0 | 0 | 0 | 0 | 0 | 1 | 0 | 2/8 |  |
| Kramska et al., 2022 | 1 | 0 | 1 | 1 | 1 | 0 | 1 | 1 | 6/8 |  |
| Myers et al., 2014 | 1 | 0 | 1 | 1 | 0 | 0 | 1 | 1 | 5/8 |  |
| Pennington et al., 2015 | 1 | 0 | 1 | 0 | 0 | 0 | 1 | 0 | 3/8 |  |
| **Cohort Studies** | | | | | | | | | | |
|  | **Selection** | **Comparability** | **Outcome** | **Total Score (0-9)** |  |  |  |  |  |  |
|  | Representativeness of the exposed cohort  a) truly representative of the average (FND patient) in the community*  b) somewhat representative of the average (FND patient) in the community*  c) selected group of users eg nurses, volunteers  d) no description of the derivation of the cohort | Selection of the non-exposed cohort  a) drawn from the same community as the exposed cohort*  b) drawn from a different source  c) no description of the derivation of the non-exposed cohort | Ascertainment of exposure  a) secure record (eg surgical records)*  b) structured interview*  c) written self-report  d) no description | Demonstration that outcome of interest was not present at start of study  a) yes*  b) no | Comparability of cohorts on the basis of the design or analysis  a) study controls for age (select the most important factor)* | Comparability of cohorts on the basis of the design or analysis (other factors)  b) study controls for any additional factor* (this criteria could be modified to indicate specific control for a second important factor) | Assessment of outcome  a) independent blind assessment*  b) record linkage*  c) self-report  d) no description | Was follow-up long enough for outcomes to occur  a) yes* (e.g., only one time point necessary)  b) no | Adequacy of follow up of cohorts  a) complete follow up - all subjects accounted for*  b) subjects lost to follow up unlikely to introduce bias - small number lost - > ____ % (select an adequate %) follow up, or description provided of those lost)*  c) follow up rate < ____% (select an adequate %) and no description of those lost  d) no statement |  |
| Schwilk et al., 2021 | 1 | 0 | 1 | 1 | 1 | 1 | 0 | 1 | 1 | 7/9 |

**Supplementary Table 2. Performance validity test scores**

| **Authors, year, location** | **Performance validity tests** |
| --- | --- |
| Binder et al., 1998 | Portland Digit Recognition Test (PDRT)  FS: 50.5 (11.5)  ES: 58.8 (7.5)  t(60)=3.42, p<.001 |
| Drane et al., 2006 | Word Memory Test (WMT)  FS:  Pass: n=19  DDI (SD): 33.1 (176)  Fail: n=18  DDI (SD): 73.3 (20.6)  ES:  Pass: n=34  DDI (SD): 49.4 (24.2)  Fail: n=3  DDI (SD): 60.1 (37.6)  Patients who failed the WMT: >11 times more likely to be diagnosed with PNESs than with ESs (OR=11.33; p<0.001) |
| Heintz et al., 2013 | Amsterdam Short Term Memory Test (ASTM)  Cut-off score = 85  ASTM performance below cut-off point (yes/no)  FMS: 5/21  GTS: 3/13  HC: 1/21 |
| Hill et al., 2003 | TOMM Trial 1  FS: 45.53 (5.76)  TLE: 45.72 (4.14)  TOMM Trial 2  FS: 48.40 (4.51)  TLE: 49.34 (1.36)  TOMM Retention Trial  FS: 48.25 (4.76)  TLE: 49.02 (2.13) |
| Kramska et al., 2022 | RBANS Effort Index (EI)  1.20 (1.926)  Min = 0  Max = 10  EI<3 n=225 EI>3 n=25 |
| McWhirter et al., 2021 | Medical Symptom Validity Test (MSVT)  FCD  Pass/valid profile = 16 (52%)  Invalid profile = 7 (23%)  Dementia profile = 8 (26%)  Not FCD  Pass/valid profile = 7 (41%)  Invalid profile = 3 (18%)  Dementia profile = 7 (41%) |
| O’Brien et al., 2015 | Only participants who passed the MSVT had their data included in the analyses. |
| Pennington et al., 2015 | Reliable Digit Span (n=9) or Reliable Digit Span & Test of Memory Malingering (n=10)  Of 19 subjects tested, 17 passed at least one validity test.  9/19 patients had invalid neuropsychological results and 10/19 had scores which were an accurate reflection of their abilities. 80% of those with valid results scored in the normal range. |
| Pick et al., 2023 | There were no significant differences between FND and HCs on the Medical Symptom Validity Test, and all participants scored above the cut-off.  Immediate recall % correct: Mdn (IQR)  FND: 100.0 (0.0)  HC: 100.0 (0.0)  W=136.0, p=1.00, r=0.00  Delayed recall % correct: Mdn (IQR)  FND: 100.0 (0.0)  HC: 100.0 (0.0)  W=120.0, p=0.18, r=0.24  Consistency %: Mdn (IQR)  FND: 100.0 (0.0)  HC: 100.0 (0.0)  W=120.0, p=0.18, r=0.24  Paired associates % correct: Mdn (IQR)  FND: 100.0 (0.0)  HC: 100.0 (0.0)  W=120.5, p=0.34, r=0.17  Free recall % correct: M (SD)  FND: 83.1 (12.8)  HC: 92.6 (11.1)  t(31)=-.12, p=0.91, g=0.04  MSVT pass: n (%)  FND: 16 (100)  HC: 17 (100) |
| Salinsky et al., 2020 | Test of Memory Malingering (TOMM)  TOMM (T1, T2 and retention trials)  Invalid TOMM performance = score of ≤ 39 on trial 1, or ≤ 44 on trial 2 or the retention trial.  FS  Valid: 56 (74.7%)  Invalid: 19 (25.3%)  ES  Valid: 58 (89.2%)  Invalid: 7 (10.8%) |
| Strutt et al., 2011 | Test of Memory Malingering (TOMM)  Suspect Effort  TOMM Trial 1  FS: 47.4 (2.85)  LTLE: 48.6 (1.25)  TOMM Trial 2  FS: 49.2 (2.08)  LTLE: 49.9 (0.33) |
| Tyson et al., 2018 | Base rates of PVT failure  Test of Memory Malingering (TOMM)  Liberal cut-off  FS: 37.5%  ES: 34.5%  Conservative cut-off  FS: 12.5%  ES: 6.9%  Vocabulary minus Digit Span (VC-DS)  ≥ 3  FS: 25.5%  ES: 20.6%  ≥ 5  FS: 25.8%  ES: 10.3%  Reliable Digit Span (RDS)  ≤ 7  FS: 27.3%  ES: 45.1%  ≤ 6  FS: 12.1%  ES: 29.6%  Digit Span (DS)  ≤ 6  FS: 21.9%  ES: 45.1 %  ≤5  FS: 15.6 %  ES: 29.6 %  Forced Choice Recognition trial of CVLT  ≤15  FS: 32.3%  ES: 12.3%  ≤14  FS: 25.8%  ES: 6.25%  FAS  ≤33  FS: 24.2%  ES: 50.7%  ≤32  FS: 21.2%  ES: 44.9%  Boston Naming Test  ≤37  FS: 25.0%  ES: 67.6%  ≤35  FS: 25.0%  ES: 62.0%  Complex Ideational Material  ≤29  FS: 10.0%  ES: 30.9%  ≤23  FS: 6.7%  ES: 19.1%  Logical Memory Recognition trial  ≤20  FS: 13.3%  ES: 17.9%  ≤18  FS: 6.7%  ES: 10.4% |
| Vechetova et al., 2022 | 27 out of 30 patients (90%) performed above threshold on the PVTs: Reliable Digit Span (RDS), Delayed Matching-to-Sample Task 48 (DMS-48), AVLT forced choice recognition (AVLT FCR)  AVLT FCR  FS: 13.89 (1.4)  HC: 13.87 (0.35)  p=.80, d=.06  DMS-48  FS: 46.48 (2.0)  HC: 47.5 (1.0)  p=.025, d=.64  RDS  FS: 8.30 (1.0)  HC: 9.07 (1.5)  p=.030, d=.58 |

**Supplementary Figure 1. Funnel Plots**

*Notes*. BNT = Boston Naming Test; ES = epilepsy; FND = functional neurological disorder; FS = functional seizures; HC = healthy controls; LMD = Logical Memory Delayed; LMI = Logical Memory Immediate; TMT-B = Trail Making Task-B.

**Supplementary Table 3. Study characteristics and cognitive tests used.**

| **Authors, year, location** | **FND sample (type, size, age [M/SD], gender)** | **Control sample**  **(type, size, age [M/SD], gender)** | **Neurocognitive test(s) used** | **Neurocognitive domain being measured** |
| --- | --- | --- | --- | --- |
| Almis et al., 2013  Turkey | Functional neurological disorder (FND)  N = 22  Age: 24.27 (7.86)  22F:0M | Healthy controls (HC)  N = 22  Age: 24.59 (7.98)  22F:0M | Wechsler Memory Scale-Revised (WMS-R) (1, 2)  Stroop Test (ST) (2, 3)  Cancellation Test (CT) (2, 4) | Memory  Working memory and attention  Attention |
| Ball et al., 2021  United Kingdom | Functional cognitive disorder (FCD)  N = 21  Age: 58.3 (12.6)  10F:11M | HC  N = 25  Age: 60.8 (5.8)  18F:7M  Neurodegenerative Mild Cognitive Impairment (nMCI)  N = 17  Age: 72.1 (11.7)  8F:9M | Trail Making Test Part B (TMT-B) (5)  Hopkins Verbal Learning Test- Revised (HLVT-R) (6) | Executive control  Verbal learning and memory |
| Bharambe & Larner, 2018  United Kingdom | FCD  N = 51  Age: 54.6 (13.0)  Range = 22-82  23F:28M | Cognitive disorders  (CD)  N = 38  Age: 71.3 (8.1)  Range = 53-88  20F:18M | Mini-Addenbrookes Cognitive Examination (M-ACE) (7) | Cognitive impairment |
| Bhome et al., 2019  United Kingdom | FCD  N = 47  Age: 52.43 (12.85)  26F:21M |  | Wechsler Adult Intelligence Scale-III (WAIS-III) (8)  National Adult Reading Test (NART) (9)  Recognition Memory Test (RMT) (10)  Symbol Digit Modalities Test (SDMT) (11)  Visual Object and Space Perception battery (VSOP) (12) | Verbal and non-verbal IQ, intellectual functioning  Visual and verbal memory  Processing speed  Visuospatial |
| Binder et al., 1998  United States | Functional seizures (FS)  N = 30  Age: 36.0 (9.8)  14F:16M | Epileptic seizures (ES)  N = 42  Age: 35.2 (11.5)  15F:27M  HC  N = 47  Age: 36.1 (12.7)  16F:31M | Wechsler Adult Intelligence Scale (WAIS-R) (13)  Wisconsin Card Sorting Test (WCST) (14)  Trail Making Task Part A and B (TMT-A, TMT-B) (5, 15)  Controlled Oral Word Association Test (COWAT) (16, 17)  Continuous Visual Memory Test (CVMT) (18, 19)  Rey Auditory Verbal Learning Test (Rey AVLT) (20, 21)  Wechsler Memory Scale-Revised (WMS-R) (1, 22)  Wide Range Achievement Test-Revised (WRAT-R) Reading (23, 24)  Grooved Pegboard (25)  Boston Naming Test (BNT) (26) | Intellectual functioning  Flexibility  Visual attention and task-switching  Verbal fluency  Visual learning and memory  Learning and verbal memory  Memory  Language  Performance speed  Naming/language |
| Borelli et al., 2022  Brazil | FCD  N = 146  Age: 66.2 (9.4)  100F:46M | Mild cognitive impairment (MCI)  N = 51  Age: 73.7 (8.4)  29F:22M  Neurodegenerative disorders (ND)  N = 299  Age: 72.4 (10.4)  174F:125M | Mini-Mental State Examination (MMSE) (27) | Attention and orientation, language, memory, recall |
| Bortz et al., 1995  United States | FS  N = 18  Age: 33.6 (11.3)  13F:5M | Left temporal foci (LTF)  N = 12  Age: 36.8 (8.6)  7F:4M  Right temporal foci (RTF)  N = 11  Age: 31.1 (12.2)  8F:3M | California Verbal Learning Test (CVLT) (28) | Verbal learning and memory |
| Brown et al., 1991  United Kingdom | FS  N = 23  Age: 37.1 (12.3)  Range = 19-59  18F:5M | ES  N = 25  Age: 33.2 (8.2)  Range = 18-46  16F:9M | Wechsler Adult Intelligence Scale-Revised (WAIS-R): Information, Arithmetic, Block Design, Similarities, Proverb Interpretation, Digit Span (29)  Boston Naming Test (BNT) (26); FAS Controlled Word Association Test (17)  Benton Judgment of Line Orientation (JLO) (30, 31)  Benton Visual Retention Test (BVRT) (32); Wechsler Memory Scale (WMS): Logical Memory, Paired Associates (33) | Language-related subskills, judgement and reasoning, visuospatial/constructive abilities, attention and memory  Language-related subskills  Visuospatial/constructive abilities  Attention and memory |
| Brown et al., 2014  United Kingdom | FND  N = 21  Age: 38.10 (11.57)  14F:7M | HC  N = 36  Age: 38.94 (12.44)  22F:14M | National Adult Reading Test (NART) (9)  Wechsler memory Scale-third edition (WMS-III): Logical Memory (34)  Trail Making Task (TMT) (15)  Stroop Colour-Word Test (35) | Intellectual functioning  Logical memory  Visual attention and processing speed (part A), set shifting ability (part B)  Selective attention, cognitive flexibility, processing speed |
| Caceres et al., 2021  Spain | FS  N = 24  Age: 36.6 (10.3)  18F:6M | Temporal lobe epilepsy (TLE)  N = 24  Age: 37.2 (10.8)  14F:10M | Wechsler Adult Intelligence Scale (WAIS-III): Vocabulary, Information, Block Design, Digit Span Forward, Digit Span Backward (36, 37)  Rey Auditory Verbal Learning Test (RAVLT) Trial 1 and Trial 5 (21, 38)  Trail Making Test Part A (TMT-A) and TMT-B (5)  Grooved Pegboard dominant hand score (25) Wechsler Memory Scale-III (WMS-III): Visual Reproduction, Faces (39)  Boston Naming Test (BNT) (26)  Semantic Verbal Fluency test (animals) (40) | Intellectual functioning, attention and psychomotor speed, executive functioning  Attention and psychomotor speed, verbal memory, executive functioning  Visual memory  Naming/Language  Executive functioning |
| Criswell et al., 2010  United States | Functional motor symptoms (FMS)  N = 13  Age: 49 (11.50)  10F:3M | Idiopathic Parkinson disease (IPD)  N = 101  Age: 69 (9.02)  44F:57M  Essential tremor (ET)  N = 49  Age: 61 (16.02)  34F:15M  Dystonia  N = 32  Age: 58 (10.28)  27F:5M  HC  N = 130  Age: 64 (11.09)  83F:47M | Finger tapping test (FTT) (41, 42) | Fine motor skills |
| de Vroege et al., 2021  Netherlands | FND  N = 29  Age: 42.4 (13.8)  23F:6M | Somatic symptom and related disorders (SSRD)  N = 289  Age: 42.1 (13.3)  174F:115M | Wechsler Adult Intelligence Scale (WAIS-IV): Digit Symbol Substitution, Digit Span (43)  Trail Making Test (TMT-A) and TMT-B (44)  Stroop Color-Word Test (SCWT) Card 1, Card 2, Card 3 (35)  Behavioural Assessment of Dysexecutive Syndrome (BADS): Key Search, Zoo Map, Rule Shift Cards (45)  Semantic and phonological verbal fluency tests (46); Rey Auditory Verbal Learning Test (RAVLT) (47); Rivermead Behavioural Memory Test (RBMT) (48)  Rey-Osterrieth Complex Figure Test (ROCFT) (49)  Boston Naming Test (BNT) (26) | Information processing speed, divided attention, working memory, attention  Executive functioning: planning, cognitive flexibility  Executive functioning: verbal fluency  Verbal memory  Visual memory and visuospatial construction  Language |
| Demartini et al., 2014    United Kingdom | FMS  N = 55  Age: 43 (10.55)  42F:13M | Organic movement disorders (OMD)  N = 33  Age: 45.70 (14.64)  23F:10M    HC  N = 34  Age: 42.18 (11.32)  23F:11M | Reading the Mind in the Eyes Test (RMET) (50) | Social cognition and emotion recognition |
| Demartini et al., 2019  Italy | FMS  N = 10  Age: 47.10 (17.00)  9F:1M | HC  N = 10  Age: 44.3 (12.85)  9F:1M | Mini-Mental State Examination (MMSE) (27) | Attention and orientation, language, memory, recall |
| Demir et al., 2013  Turkey | FND+psychiatric comorbidities  N = 43  Age: 31.09 (11.65)  37F:6M | Psychiatric diagnoses (PD)  N = 44  Age: 31.86 (10.78)  35F:9M  HC  N = 43  Age: 36.02 (11.29)  36F:7M | Serial Digit Learning Test (SDLT) (2, 51)  Auditory Verbal Learning Test (AVLT) (21, 52)  Wechsler Memory Scale (WMS) (33)  Stroop Color Word Interference Test (ST) (2, 35)  Benton Judgment of Line Orientation Test (BJLOT) (2, 30)  Cancellation Test (2, 53) | Learning and memory  Verbal learning and memory  Memory  Executive functioning  Visuospatial perception  Attention |
| Drane et al., 2006  United States | FS  N = 43  Age: 40.6 (10.2)  34F:9M | ES  N = 41  Age: 36.9 (14.4)  19F:22M | Neuropsychological Battery for Epilepsy (54) | Neurocognitive functioning |
| Dunbar et al., 2021  United States | FS  N = 22  Age: 42.1 (15.1)  16F:6M | ES  N = 54  Age: 36.9 (12.3)  32F:22M | Conners Continuous Performance Test-III (CPT-III) (55) | Sustained attention |
| Giugno et al., 2023  Italy | FS  N = 22  Age: 40.0 (13.10)  18F:4M | TLE  N = 28  Age: 37.82 (9.31)  22F:6M | Rey Auditory Verbal Learning Test (RAVLT) (20, 21)  FAS (17)  Weigl Color-Form Sorting Test (56)  Digit Span Forwards and Backward (57)  Rey-Osterrieth Complex Figure Test (ROCFT) (49) | Verbal learning  Lexical stock  Executive function  Short-term and working memory  Visuospatial functions |
| Gursoy et al., 2021  Turkey | FS  N = 28  Age: 36.86 (8.39)  22F:6M | ES  N = 28  Age: 34.04 (8.88)  19F:9M    HC  N = 28  Age: 35.11 (7.19)  20F:8M | Reading the mind in the eyes test (RMET) (50, 58) | Social cognition and emotion recognition |
| Hamouda et al., 2021  Germany | FS  N = 40  Age: 35.82 (12.58)  30F:10M | HC  N = 40  Age: 36.48 (12.51)  30F:10M | Attention Network Task (ANT) (59)  Wechsler Adult Intelligence Scale (WAIS-IV): Digit Span Forward and Backward (43)  Trail Making Tests A and B (TMT-A, TMT-B) (15) | Alerting, orienting and executive function  Working memory  Attentional executive control and set-shifting abilities |
| Heintz et al., 2013  Netherlands | FMS  N = 26  Age: 53.6 (13.8)  8F:18M | Gilles de la Tourette syndrome (GTS)  N = 13  Age: 33.4 (11.8)  3F:13M  HC  N = 22  Age: 50.6 (13.0)  9F:13M | Trail Making Test Part A (TMT-A) and Trail Making Test Part B (TMT-B) (60); Stroop Task: word reading, colour naming, colour-word interference condition (61); Dutch Intelligence Test: Category and phonemic fluency tasks (62)  Dutch version of Rey Auditory Verbal Learning Test (AVLT) (21); Rivermead Behavioural Memory Test (RBMT): prose recall task (63); Wechsler Memory Scale-III (WMS-III): Visual Reproduction (34)  Vienna Test System (VTS): subtests S1 and S3 (64) | Attention, executive functioning  Memory  Processing speed/reaction time |
| Hill et al., 2003  United States | FS  N = 57  Age: 36.84 (12.02)  41F:16M | TLE  N = 48  Age: 37.13 (10.81)  23F:25M | Wechsler Adult Intelligence Scale–Revised (WAIS-R) (29)  California Verbal Learning Test (CVLT) (28)  Wechsler Memory Scale–Revised (WMS-R) (1) | Intelligence  Declarative memory  Memory |
| Hill & Gale, 2011  United States | FS-Non-motor  N = 70  Age: 40.9 (15.2)  53F:17M  FS-Motor  N = 103  Age: 39.8 (12.5)  74F:29M | TLE  N = 180  Age: 38.7 (13.0)  107F:73M | Wechsler Abbreviated Scale of Intelligence (WASI) (65)  Wechsler Adult Intelligence Scale-III (WAIS-III): Digit Span (66)  Halstead Reitan Trail Making Test, Part A and Part B (TMT-A; TMT-B) (60)  Boston Naming Test (BNT) (26)  Brief Visuospatial Memory Test – Revised (BVMT-R) (67)  Rey Auditory-Verbal Learning Test (RAVLT) (20, 21) | Intellectual functioning  Working memory  Psychomotor speed, visual search, attention  Naming/language  Visuospatial memory  Verbal memory |
| Huys et al., 2020  United Kingdom | FMS  N = 30  Age: 47.5 (no SD)  Range: 21-79  17F:13M | Organic movement disorder (OMD)  N = 30  Age: 48.0  Range: 21-77  15F:15M  HC  N = 30  Age: 44.7  Range: 24-79  16F:14M | Attention Network Test (ANT) (59) | Alerting, orienting and executive networks efficiencies |
| Irorutola et al., 2020    Germany | FS  N = 41  Age: 35.98 (11.99)  32F:9M | HC  N = 41  Age: 35.90 (12.42)  32F:9M | Reading the Mind in the Eyes Test (RMET) (50) | Social cognition and emotion recognition |
| Kramska et al., 2022  Czech Republic | FS  N = 250  Age: 38.32 (13.23)  186F:64M |  | Repeatable Battery for the Assessment of Neuropsychological Status (RBANS, Czech Research version) (68) | Immediate Memory, Visuospatial/Constructional, Language, Attention, and Delayed Memory |
| Leon-Sarmiento et al., 2019  United States | FMS  N = 35  Age: 49.0 (13.2)  25F:10M | HC  N = 35  Age: 49.8 (12.5)  25F:10M  Parkinson’s disease (PD)  N = 17  Age: Not reported | Picture Identification Test (PIT) (69) | Word recognition |
| Lloyd et al., 2022  Australia | FS  N = 59  Age: Not reported  40F:19M | ES  N = 202  Age: Not reported  111F:101M | Neuropsychiatry Unit Cognitive Assessment Tool (NUCOG) (70) | Attention, memory, language, executive, and visuospatial function |
| Matthews et al., 2020  Australia | FMS  N = 20  Age: 45.6 (17.7)  Range: 20-69  13F:7M | HC  N = 20  Age: 41.7 (16.9)  Range: 21-68  14F:6M  Organic motor disorders (OMD)  N = 20  Age: 63.7 (10.9)  Range: 34-82  8F:12M | Montreal Cognitive Assessment (MoCA) (71) | Short-term memory, attention, executive function, visuospatial abilities, working memory, language, abstract reasoning, orientation |
| McWhirter et al., 2021  United Kingdom | FCD  N = 31  Age: 63.2 (14.3)  18F:13M | Not FCD  N = 18  Age: 81.8 (5.87)  8F:10M | Montreal Cognitive Assessment (MoCA) (71)  Clinical Addenbrookes Cognitive Examination iii (ACEiii) (72) | Short-term memory, attention, executive function, visuospatial abilities, working memory, language, abstract reasoning, orientation  Memory, visual perception, attention, language, orientation, visuospatial skills |
| Myers et al., 2014  United States | FS no trauma  N = 17  Age: 35.29 (12.1)  17F:0M | FS+PTSD  N = 17  Age: 45.53 (10.74)  17F:0M  FS+trauma  N = 29  Age: 37.65 (11.86)  22F:7M | Wechsler Abbreviated Scale of Intelligence (WASI) (65)  Delis-Kaplan Executive Function System (D-KEFS): Trail Making, Color-Word Interference (73)  Wechsler Memory Scale (WMS-III) (34); Continuous Visual Memory Test (CVMT) (19)  California Verbal Learning Test-II (CVLT-II) (74)  Boston Naming Test (BNT) (26) | Intellectual functioning  Shifting, inhibition  Memory  Verbal learning and memory  Naming/language |
| O’Brien et al., 2015  Ireland | FS  N = 19  Age: 30.0 (8.8)  13F:6M | HC  N = 19  Age: 29.7 (7.0)  13F:6M | Wechsler Abbreviated Scale of Intelligence (WASI) (36, 65)  Wechsler Test of Adult Reading (WTAR) (75)  Cambridge Neuropsychological Test Battery (CANTAB)*: Intra/Extradimensional Shift Task, Rapid Visual Processing (RVP) Task, Stockings of Cambridge (SOC) Task, Spatial Working Memory (SWM) Task (76)  *completed after passing the Medical Symptom Validity Test (MSVT) of effort | Intelligence  Level of intellectual functioning  Attention, planning efficiency and memory, executive functioning |
| O’Malley et al., 2020  United Kingdom | FCD  N = 15  Age: 54.9 (4.1)  40% male | Alzheimer’s disease (AD)  N = 15  Age: 67.8 (4.2)  66.7% male  MCI  N = 15  Age: 63.4 (4.2)  66.7 % male  HC  N = 15  Age: 69.5 (4.0)  40% male | Mini Mental State Examination (MMSE) (27)  Addenbrooke’s Cognitive Examination-Revised (ACE-R) (77) | Attention and orientation, language, memory, recall  Cognitive impairment |
| Ozer Celik et al., 2015  Turkey | FS  N = 20  Age: 28.85 (8.99)  18F:2M | ES  N = 11  Age: 28.82 (13.14)  4F:7M  HC  N = 20  Age: 31.05 (6.985)  15F:5M | Öktem Verbal Memory Processes Test (VMPT) (78) | Verbal learning and memory |
| Pennington et al., 2015  United Kingdom | FCD  N = 23  Age: Not reported, <60 years  15F:8M |  | Montreal Cognitive Examination (MoCA) (71) | Short-term memory, attention, executive function, visuospatial abilities, working memory, language, abstract reasoning, orientation |
| Pennington et al., 2019  United Kingdom | FCD  N = 21  Age: 58.3 (no SD provided)  10F:11M | MCI  N = 17  Age: 72.1 (no SD provided)  8F:9M  HC  N = 25  Age: 60.8 (no SD provided)  18F:7M | Montreal Cognitive Assessment (MoCA) (71) | Short-term memory, attention, executive function, visuospatial abilities, working memory, language, abstract reasoning, orientation |
| Pick, Mellers, & Goldstein, 2016  United Kingdom | FS  N = 40  Age: 40 (23) *Median(IQR)  32F:8M | HC  N = 43  Age: 36 (20) *Median(IQR)  35F:8M | Wechsler Abbreviated Scale of Intelligence (WASI): Vocabulary, Matrix Reasoning (65)  Benton Facial Recognition Test (BFRT) (79)  Wechsler Memory Scale-Third Edition (WMS-III): Faces I (34) | Intellectual functioning  General perceptual processing of facial stimuli  Short-term memory for facial stimuli |
| Pick, Mellers, & Goldstein, 2018a  United Kingdom | FS  N = 39  Age: 37.9 (13.2)  31F:8M | HC  N = 42  Age: 37.3 (11.8)  35F:7M | Wechsler Abbreviated Scale of Intelligence (WASI): Vocabulary, Matrix Reasoning (65)  Visual Object and Space Perception Battery (VOSP): Object Decision (OD) (12)  Wechsler Memory Scale-Third Edition (WMS-III): Family Pictures I (34) | Intellectual functioning  General object perception/recognition  Immediate memory for complex visual scenes |
| Pick, Mellers, & Goldstein, 2018b  United Kingdom | FS  N = 38  Age: 41.5 (22.5) *Median(IQR)  30F:8M | HC  N = 43  Age: 36 (20)  *Median(IQR)  35F:8M | Wechsler Abbreviated Scale of Intelligence (WASI): Vocabulary, Matrix Reasoning (65)  Stroop test (80)  Benton Facial Recognition Test (BFRT) (79) | Intellectual functioning  Executive functioning  General perceptual processing of facial stimuli |
| Pick et al., 2023  United Kingdom | FND  N = 16  Age: 36.1 (10.8)  12F:4M | HC  N = 17  Age: 39.0 (11.0)  13F:4M | Wechsler Abbreviated Scale of Intelligence – 2^nd^ edition (WASI-II) (81)  Cambridge Neuropsychological Test Automated Battery (CANTAB) Connect: Motor Screening, Reaction Time, Rapid Visual Information Processing, Spatial Span, Intra-Extra Dimensional Set Shift, Stop Signal Task, Emotional Bias Task, Emotion Recognition Test (82) | Intellectual functioning  Sensorimotor speed, cognitive and motor response speed, attention, working memory, attentional set-shifting, cognitive flexibility, visual discrimination, response inhibition, social cognition |
| Prigatano & Kirlin, 2009  United States | FS  N = 23  Age: 42.87 (12.41)  17F:6M | ES  N = 22  Age: 40.77 (13.40)  11F:11M | Rey Auditory Verbal Learning Test (RAVLT): Delayed Recall (21)  Brief Visuospatial Memory Test-Revised (BVMT-R): Delayed Recall (67)  BNI Screen for Higher Cerebral Functions (BNIS): Memory subscale (83)  Trail Making Test Part B (TMT-B) (44); Wechsler Adult Intelligence Scale III (WAIS-III): Digit Span (66)  Wechsler Abbreviated Scale of Intelligence (WASI): Verbal IQ, Performance IQ (65)  Boston Naming Test (BNT) (26) | Memory  Executive functioning, working memory  Word-finding ability, visuospatial abilities |
| Sackellares & Sackellares, 2001  United States | FS  N = 40  Age: 32.8 (no SD reported)  31F:9M | HC  N = 40  Age: 33.2 (no SD reported)  Range: 18-50 | Halstead-Reitan Neuropsychological Test Battery: Finger Oscillation, Grip Strength (84)  Wechsler Adult Intelligence Scale–Revised (WAIS-R) (29) | Motor speed and strength  Intellectual functioning |
| Salinsky et al., 2020  United States | FS  N = 75  Age: not reported  ?F:?M | ES  N = 65  Age: not reported  ?F:?M | Repeatable Battery for the Assessment of Neuropsychological Status (RBANS) Total Scale index (68) | Immediate memory, visuospatial/constructional, language, attention, delayed memory |
| Schwilk et al., 2021  Germany | FCD  N = 28  Age: 54.7 (6.8)  ?F:?M |  | German version of the Auditory Verbal Learning Test (VLMT) (85)  “Zahlenverbindungstest” (ZVT) (86)  Mehrfachwahl-Wortschatztest (MWT-B) (87) | Declarative memory  Information processing speed  Verbal intelligence |
| Silveri et al., 2022  Italy | FMS  N = 18  Age: 61.17 (18.58)  13F:5M | HC 1  N = 14  Age: 64.86 (10.98)  7F:7M  HC 2  N = 14  Age: 61.93 (12.68)  7F:7M | Mini-Mental State Examination (MMSE) (27)  Raven’s Coloured Progressive Matrices (88)  Reading the Mind in the Eyes Test (RMET) (50) | Attention and orientation, language, memory, recall  Non-verbal intelligence  Social cognition and emotion recognition |
| Simani et al., 2020a  Iran | FS  N = 30  Age: 30.68 (10.44)  20F:10M | Genetic generalized epilepsy (GGE)  N = 30  Age: 27.36 (7.5)  20F:10M  HC  N = 32  Age: 31.87 (8.05)  18F:14M | Integrated Visual and Auditory [Continuous Performance Test](https://www.sciencedirect.com/topics/medicine-and-dentistry/continuous-performance-test) (IVA-CPT) (89)  [Wechsler Adult Intelligence Scale](https://www.sciencedirect.com/topics/medicine-and-dentistry/wechsler-adult-intelligence-scale) (WAIS) (65) | Attention and inhibitory control  General intelligence |
| Simani et al., 2020b  Iran | FS  N = 35  Age: 31.68 (10.03)  23F:12M | Idiopathic generalized epilepsy (IGE)  N = 35  Age: 28.85 (8.71)  22F:13M  HC  N = 35  Age: 31.94 (7.56)  20F:15M | Integrated Visual and Auditory [Continuous Performance Test](https://www.sciencedirect.com/topics/medicine-and-dentistry/continuous-performance-test) (IVA-CPT) (89)  [Wechsler Adult Intelligence Scale](https://www.sciencedirect.com/topics/medicine-and-dentistry/wechsler-adult-intelligence-scale) (WAIS) (65) | Attention and inhibitory control    General intelligence |
| Slater et al., 1995  United States | FS  N = 22  Age: 33.875 (10.82)  1F:2.2M | ES  N = 27  Age: 34.3 (8.026)  1F:1M | Wechsler Adult Intelligence Scale-Revised (WAIS-R): Information, Arithmetic, Similarities, Proverb Interpretation, Block Design, Digit Span (29)  Boston Naming Test (BNT) (26)  FAS Controlled Word Association Test (16)  Judgment of Line Orientation (JLO) (31); Benton Visual Retention Test (BVRT) (32); Wechsler Memory Scale (WMS): Visual Reproduction, Logical Memory Passage, Paired Associates (33) | Language, judgment and reasoning, visuospatial/constructive abilities, attention and memory  Visuospatial/constructive abilities  Attention and memory |
| Strutt et al., 2011  United States | FS  N = 33  Age: 38.5 (13.4)  33F | LTLE  N = 25  Age: 35.0 (11.4)  25F | Sattler's IQ Estimate (Sattler's Equation = V + A + DS + S)  Wechsler Adult Intelligence Scale, Third Edition (WAIS-III): Digit Span, Arithmetic, Letter–Number Sequencing, Working Memory Index, Similarities (8)  Wechsler Memory Scale-III (WMS-III): Spatial Span, Mental Control, Logical Memory I and II, Verbal Paired Associates I and II, Visual Reproduction I and II (34)  Revised Comprehensive Norms for the Expanded Halstead–Reitan Battery: Trail Making Test Part A (TMT-A), Boston Naming Test (BNT), Letter Fluency (FAS), Semantic Fluency (Animals), Trail Making Test Part B (TMT-B) (90) | Intellectual functioning  Attention, working memory, executive functioning  Attention, working memory, information processing speed, verbal memory, visual memory  Information processing speed, language, executive functioning |
| Tyson et al., 2018  United States | FS  N = 33  Age: 39.5 (13.0)  18F:15M | ES  N = 72  Age: 35.7 (11.3)  39F:33M | Animal Fluency (Animals), Letter Fluency (FAS) (16, 17)  Wechsler Adult Intelligence Scale (WAIS): Arithmetic, Block Design, Coding, Digit Span, Information, Matrix Reasoning, Similarities, Symbol Search, Vocabulary (91)  Boston Naming Test (BNT) (26)  California Verbal Learning Test – Second Edition (CVLT-II) (74)  Complex Ideational Material (CIM) (92, 93)  Delis-Kaplan Executive Function System (D-KEFS) (73)  Finger Tapping Test (FTT) (42)  Wechsler Memory Scale (WMS): Logical Memory (Immediate and Delayed Recall) (33)  Wide Range Achievement Test – Reading (WRAT) (23, 24)  Wisconsin Card Sorting Test (WCST) (14) | Verbal fluency  Auditory working memory, Visuospatial and construction skills, processing speed, auditory working memory, general fund of knowledge, visual reasoning, verbal reasoning, processing speed, word knowledge  Object naming/word finding  Word list learning/memory  Receptive language  Executive function  Manual dexterity  Story memory  Single word reading  Concept formation/executive function |
| Vechetova et al., 2022  Czech Republic | FMS  N = 30  Age: 45.3 (9)  21F:9M | HC  N = 30  Age: 45.3 (10)  21F:9M | Mini-Mental State Examination (MMSE) (94)  Trail Making Test Part A (TMT-A) and TMT-B (95)  Stroop Task: word reading, colour naming, colour-word interference (96); Wechsler Adult Intelligence Scale-III (WAIS-III): Digit Span Forward, Digit Span Backward (97)  Phonemic fluency tasks using letters K, P, S (98)  Czech version of the N-back task (99)  Rey Auditory Verbal Learning Test (AVLT) (100, 101)  Rey-Osterreith Complex Figure Test (ROCFT) (101, 102)  Semantic verbal fluency tests (categories animals, vegetables) (98); 15-item Boston Naming Test (103)  Visual Object and Space Perception Battery (VOSP): Number Location subtest (104, 105) | Attention and orientation, language, memory, recall  Attention, executive functioning, executive functioning, working memory  Executive functioning  Working memory  Short-term memory  Visuospatial memory, visuospatial functions  Speech and language  Visuospatial functions |
| Voon et al., 2013  United Kingdom | FMS  N = 30  Age: 47.98 (13.61)  20F:10M | HC  N = 30  Age: 50.62 (12.80)  20F:10M | Conner’s Continuous Performance Test II (106)  Wechsler Test of Adult Reading (WTAR) (75)  Wechsler Adult Intelligence Scale (WAIS): Symbol Search Test, Digit Symbol Test (66)  Hopkins Verbal Learning Test (HVLT) (107); Brief Visuospatial Memory Test (BVMT) (108)  Delis-Kaplan Executive Function System (D-KEFS): Tower Test, Verbal Fluency Test, Color-Word Interference Test (73)  Boston Naming Test (BNT) (26)  RBANS: Judgment of Line Orientation (68) | Motor response inhibition  Visual, performance and full-scale IQ  Processing speed  Memory  Planning and problem solving, verbal fluency, verbal inhibition, simultaneous processing, cognitive flexibility  Object naming  Visuospatial processing |
| Walterfang et al., 2011  Australia | FS  N = 50  33.1 (12.5)  73% female | ES  N = 87  37.6 (13.8)  59% female  Other seizures (OS)*  N = 27  Age: 34.5 (17.3)  58% female  *included other medical and psychiatric causes | Neuropsychiatry Unit Cognitive Assessment Tool (NUCOG) (70) | Attention, Memory, Language, Visuoconstructional, and Executive Function |
| Wilkus & Dodrill 1989  United States | FS  N = 25  Age: 28.12 (10.35)  21F:4M | Partial epileptic (PE)  N = 25  Age: 28.20 (8.12)  21F:4M  Generalized epileptic A (GEA)  N = 25  Age: 27.80 (10.24)  21F:4M  Generalized epileptic B (GEB)  N = 25  Age: 27.88 (10.75)  21F:4M | Wechsler Adult Intelligence Scale (WAIS) (91)  Neuropsychological Battery for Epilepsy (54) | Intelligence  Cognitive functioning |
| Wilkus, Dodrill, & Thompson, 1984  United States | FS  N = 25  Age: 28.2 (10.35)  21F:4M | ES  N = 25  Age: 29.8 (9.70)  ?F:?M | Wechsler Adult Intelligence Scale (WAIS) (91)  Neuropsychological Battery for Epilepsy (54) | Intelligence  Cognitive functioning |
| Ye et al., 2020  Australia | FS  N = 85  Age: 35.0 (13)  Range: 18-74  64F:21M | ES  N = 156  Age: 40.4 (14.9)  Range: 18-77  85F:71M  Non-diagnostic group (ND)  N = 68  Age: 41.8 (15.8)  Range: 19-73  39F:29M | Neuropsychiatry Unit Cognitive Assessment Tool (NUCOG) total score (70) | Attention, memory, language, visuoconstructional, executive function |

**Supplementary References for Table 3**

1. Wechsler D. Wechsler memory scale-revised. Psychological Corporation. 1987.

2. Karakas S. BILNOT battery: research and development of neuropsychological tests. Dizayn Ofset, Ankara. 2004.

3. MacLeod CM. The Stroop task: The" gold standard" of attentional measures. Journal of Experimental Psychology: General. 1992;121(1):12.

4. Weintraub S. Mental state assessment of young and elderly adults in behavioral neurolagy. Principles of behavioral neurology. 1985:71-168.

5. Tombaugh TN. Trail Making Test A and B: normative data stratified by age and education. Archives of clinical neuropsychology. 2004;19(2):203-14.

6. Shapiro AM, Benedict RH, Schretlen D, Brandt J. Construct and concurrent validity of the Hopkins Verbal Learning Test–revised. The Clinical Neuropsychologist.

1999;13(3):348-58.

7. Hsieh S, McGrory S, Leslie F, Dawson K, Ahmed S, Butler CR, et al. The Mini-Addenbrooke's Cognitive Examination: a new assessment tool for dementia. Dement

Geriatr Cogn Disord. 2015;39(1-2):1-11.

8. Wechsler D. WAIS-III: Administration and scoring manual: Wechsler adult intelligence scale: Psychological Corporation; 1997.

9. Nelson H, Willison J. National adult reading test (NART) manual. Windsor, Bershire, UK: NFER-Nelson. 1982.

10. Warrington EK. Recognition memory test: Manual. Nfer-Nelson. 1984.

11. Smith A. Symbol digit modalities test: Western psychological services Los Angeles; 1973.

12. Warrington EK. Visual object and space perception battery. (No Title). 1991.

13. Thompson LL, Heaton RK, Grant I, Matthews CG. A comparison of the WAIS and WAIS-R using T-score conversions that correct for age, education, and sex.

Journal of clinical and experimental neuropsychology. 1989;11(4):478-88.

14. Heaton RK. Wisconsin Card Sorting Test Manual. Odessa, FL: Psychological Assessment Resources; 1981.

15. Reitan RM. Validity of the Trail Making Test as an Indicator of Organic Brain Damage. Perceptual and Motor Skills. 1958;8(3):271-6.

16. Ruff RM, Light RH, Parker SB, Levin HS. Benton controlled oral word association test: Reliability and updated norms. Archives of Clinical Neuropsychology.

1996;11(4):329-38.

17. Benton A, Hamsher dS, Sivan A. Controlled oral word association test. Archives of Clinical Neuropsychology. 1994.

18. Trahan DE, Larrabee GJ. Continuous visual memory test: Psychological Assessment Resources; 1983.

19. Larrabee GJ, Trahan DE, Curtiss G. Construct validity of the continuous visual memory test. Archives of Clinical Neuropsychology. 1992;7(5):395-405.

20. McMinn MR, Wiens AN, Crossen JR. Rey auditory-verbal learning test: Development of norms for healthy young adults. Clinical Neuropsychologist. 1988;2(1):67-

87.

21. Rey A. L'examen clinique en psychologie. 1958.

22. Elwood RW. The Wechsler Memory Scale—Revised: Psychometric characteristics and clinical application. Neuropsychology Review. 1991;2(2):179-201.

23. Witt JC. Review of the wide range achievement test-revised. Journal of Psychoeducational Assessment. 1986;4(1):87-90.

24. Spruill J, Beck B. Relationship between the WAIS-R and Wide Range Achievement Test-Revised. Educational and Psychological Measurement. 1986;46(4):1037-

40.

25. Merker B, Podell K. Grooved Pegboard Test. In: Kreutzer JS, DeLuca J, Caplan B, editors. Encyclopedia of Clinical Neuropsychology. New York, NY: Springer

New York; 2011. p. 1176-8.

26. Kaplan E, Goodglass H, Weintraub S. Boston Naming Test. Philadelphia: Lea & Febiger; 1983.

27. Folstein MF, Folstein SE, McHugh PR. “Mini-mental state”: a practical method for grading the cognitive state of patients for the clinician. Journal of psychiatric

research. 1975;12(3):189-98.

28. Delis DC, Kramer, J.H., Kaplan, E., Ober, B.A. . California verbal learning test: Adult version manual. . San Antonia, TX: The Psychological Corporation; 1987.

29. Wechsler D. Wechsler adult intelligence scale-revised manual. New York: Psychological Corporation. (No Title). 1981.

30. Benton AL, Varney NR, Hamsher KD. Visuospatial judgment. A clinical test. Arch Neurol. 1978;35(6):364-7.

31. Riccio CA, Hynd GW. Validity of Benton's Judgement of Line Orientation Test. Journal of Psychoeducational Assessment. 1992;10(3):210-8.

32. Benton AL. A VISUAL RETENTION TEST FOR CLINICAL USE. Archives of Neurology & Psychiatry. 1945;54(3):212-6.

33. Wechsler D. Wechsler memory scale. 1945.

34. Wechsler D. Wechsler Memory Scale-III. San Antonio, TX: The Psychological Corporation; 1997.

35. Stroop JR. Studies of interference in serial verbal reactions. Journal of Experimental Psychology. 1935;18:643-62.

36. Axelrod BN. Validity of the Wechsler abbreviated scale of intelligence and other very short forms of estimating intellectual functioning. Assessment. 2002;9(1):17-

23.

37. Wechsler D. WAIS-III, escala de inteligencia de Wechsler para adultos-III: manual de aplicación y corrección: TEA; 1999.

38. Miranda Jr JP, Valencia RR. English and Spanish versions of a memory test: Word-length effects versus spoken-duration effects. Hispanic Journal of Behavioral

Sciences. 1997;19(2):171-81.

39. Wechsler D. Escala de memoria de Wechsler: WMS-III: TEA; 2004.

40. Peña-Casanova J, Quinones-Ubeda S, Gramunt-Fombuena N, Quintana-Aparicio M, Aguilar M, Badenes D, et al. Spanish Multicenter Normative Studies

(NEURONORMA Project): norms for verbal fluency tests. Archives of Clinical Neuropsychology. 2009;24(4):395-411.

41. Nutt J, Carter J, Woodward W. Long-duration response to levodopa. Neurology. 1995;45(8):1613-6.

42. Ruff RM, Parker SB. Gender- and Age-Specific Changes in Motor Speed and Eye-Hand Coordination in Adults: Normative Values for the Finger Tapping and

Grooved Pegboard Tests. Perceptual and Motor Skills. 1993;76(3_suppl):1219-30.

43. Wechsler D. WAIS-IV Administration and Scoring Manual. San Antonio, TX: The Psychological Corporation; 2008.

44. Reitan RM. Trail Making Test: Manual for administration and scoring: Reitan Neuropsychology Laboratory; 1986.

45. Wilson BA, Alderman N, Burgess PW, Emslie H, Evans JJ. BADS: Behavioural assessment of the dysexecutive syndrome: Pearson London; 1996.

46. Deelman B, Koning-Haanstra M, Liebrand W, van der Burg W. A test for aphasia for auditive and verbal language use. Manual. Lisse, The Netherlands: Swets &

Zeitlinger; 1981.

47. Saan R, Deelman, B. De 15-WoordenTests A (manual). University of Groningen, Groningen, Netherlands: Department of Neuropsychology1986.

48. Wilson BA, Cockbum, J., Baddeley, A.D. The rivermead behavioural memory test. Bury St Edmunds, UK: Thames Valley Test Company; 1985.

49. Osterrieth P. The test of copying a complex figure: A contribution to the study of perception and memory. Arch Psychol. 1944;30:206-356.

50. Baron-Cohen S, Wheelwright S, Hill J, Raste Y, Plumb I. The "Reading the Mind in the Eyes" Test revised version: a study with normal adults, and adults with

Asperger syndrome or high-functioning autism. J Child Psychol Psychiatry. 2001;42(2):241-51.

51. Zangwill OL. Clinical tests of memory impairment. Proceedings of the Royal Society of Medicine. 1943;36:576-80.

52. Öktem Ö. Sözel Bellek Süreçleri Testi, Bir ön çalışma. Nöropsikoloji Arşivi 1992;29:196-206.

53. Mesulam M-M. Principles of behavioral and cognitive neurology: Oxford University Press; 2000.

54. Dodrill CB. A neuropsychological battery for epilepsy. Epilepsia. 1978;19(6):611-23.

55. Conners CK, editor Conners continuous performance test 3rd edition (Conners CPT 3) & connors continuous auditory test of attention (Conners CATA): Technical

manual2014: MHS.

56. Hobson P, Meara J, Taylor C. The Weigl Colour-Form Sorting Test: a quick and easily administered bedside screen for dementia and executive dysfunction.

International Journal of Geriatric Psychiatry. 2007;22(9):909-15.

57. Monaco M, Costa A, Caltagirone C, Carlesimo GA. Forward and backward span for verbal and visuo-spatial data: standardization and normative data from an Italian

adult population. Neurological Sciences. 2013;34:749-54.

58. Yildirim EA, Kaşar M, Güdük M, Ateş E, Küçükparlak İ, Özalmete EO. Gözlerden zihin okuma testi’nin Türkçe güvenirlik çalışması. Turk Psikiyatri Dergisi.

2011;22(3):177-86.

59. Fan J, McCandliss BD, Sommer T, Raz A, Posner MI. Testing the efficiency and independence of attentional networks. J Cogn Neurosci. 2002;14(3):340-7.

60. Reitan RM, Wolfson D. The Halstead-Reitan neuropsychological test battery: Theory and clinical interpretation: Reitan Neuropsychology; 1985.

61. Schmand B, Houx P, De Koning I. Norms for stroop color word test, trail making test, and story recall of Rivermead behavioural memory test. Amsterdam: De sectie

Neuropsychologie van het Nederlands Instituut van Psychologen. 2003.

62. Luteijn F, van der Ploeg F. Groninger intelligentie test (Dutch Intelligence test). Lisse, The Netherlands, Swets & Zeitlinger; 1998.

63. Towle D, Wilsher C. The Rivermead Behavioural memory test: remembering a short route. British journal of clinical psychology. 1989;28(3):287-8.

64. Schuhfried G. Vienna Test System, version 6.20.22. Reaction Test. 2004.

65. Wechsler D. Wechsler abbreviated scale of intelligence. 1999.

66. Wechsler D. Wechsler adult intelligence scale third edition. (No Title). 1997.

67. Benedict RH. Brief visuospatial memory test--revised: PAR; 1997.

68. Randolph C. Repeatable Battery for the Assessment of Neuropsychological Status (RBANS): Psychological Corporation San Antonio, TX; 1998.

69. Vollmecke T, Doty R, editors. Development of the Picture Identification Test (PIT)-A research companion to the University-of-Pennsylvania Smell Identification

Test (UPSIT). Chemical Senses; 1985: OXFORD UNIV PRESS WALTON ST JOURNALS DEPT, OXFORD, ENGLAND OX2 6DP.

70. Walterfang M, Siu R, Velakoulis D. The NUCOG: validity and reliability of a brief cognitive screening tool in neuropsychiatric patients. Australian & New Zealand

Journal of Psychiatry. 2006;40(11-12):995-1002.

71. Nasreddine ZS, Phillips NA, Bédirian V, Charbonneau S, Whitehead V, Collin I, et al. The Montreal Cognitive Assessment, MoCA: a brief screening tool for mild

cognitive impairment. Journal of the American Geriatrics Society. 2005;53(4):695-9.

72. Hsieh S, Schubert S, Hoon C, Mioshi E, Hodges JR. Validation of the Addenbrooke's Cognitive Examination III in frontotemporal dementia and Alzheimer's disease.

Dementia and geriatric cognitive disorders. 2013;36(3-4):242-50.

73. Delis DC, Kaplan E, Kramer JH. Delis-Kaplan executive function system. Assessment. 2001.

74. Delis DC, Kramer JH, Kaplan E, Ober BA. California verbal learning test. Assessment. 2000.

75. Wechsler D. Wechsler Test of Adult Reading: WTAR: Psychological Corporation; 2001.

76. Fray PJ, Robbins TW, Sahakian BJ. Neuorpsychiatyric applications of CANTAB. International Journal of Geriatric Psychiatry. 1996;11:329-36.

77. Mioshi E, Dawson K, Mitchell J, Arnold R, Hodges JR. The Addenbrooke's Cognitive Examination Revised (ACE-R): a brief cognitive test battery for dementia

screening. International Journal of Geriatric Psychiatry. 2006;21(11):1078-85.

78. Bosgelmez S, Yildiz M, Yazici E, Inan E, Turgut C, Karabulut U, et al. Reliability and Validity of The Turkish Version of Cognitive Assessment Interview (CAI-TR).

Klinik Psikofarmakoloji Bülteni-Bulletin of Clinical Psychopharmacology. 2015;25(4):365-80.

79. Benton AL, Sivan, A.B, Hamsher, K.D, Varney, N.R, Spreen, O. Contributions to Neuropsychological Assessment: A Clinical manual. Oxford, UK: Oxford

University Press; 1994.

80. Golden CJ, Freshwater SM, Zarabeth G. Stroop Color and Word Test Children's Version for ages 5-14: A manual for clinical and experimental uses: Stoelting; 2003.

81. Wechsler D. Wechsler Abbreviated Scale of Intelligence - Second Edition. Bloomington, MN: NCS Pearson, Inc.; 2011.

82. CambridgeCognition. Product Overview: CANTAB Connect Research v11. 10. Cambridge, UK: Cambridge Cognition Limited. 2019.

83. Prigatano G, Amin K, Rosenstein L. Administration and scoring manual for the BNI Screen for Higher Cerebral Functions. Phoenix, AZ: Barrow Neurological

Institute. 1995.

84. Boll TJ. The Halstead-Reitan Neuropsychology Test Battery. Handbook of clinical neuropsychology. 1981:577-607.

85. Helmstaedter C, Lendt M, Lux S. Verbaler Lern-und Merkfähigkeitstest: VLMT; Manual: Beltz-test; 2001.

86. Oswald WD, Roth E. Der Zahlen-Verbindungs-Test (ZVT): Hogrefe Verlag fuer Psychologie; 1987.

87. Hessler J, Jahn T, Kurz A, Bickel H. The MWT-B as an Estimator of Premorbid Intelligence in MCI and Dementia. Zeitschrift für Neuropsychologie.

2013;24(3):129-37.

88. Basso A, Capitani E, Laiacona M. Raven's coloured progressive matrices: normative values on 305 adult normal controls. Functional neurology. 1987;2(2):189-94.

89. Sanford J, Turner A. Manual for the integrated visual and auditory continuous performance test. Richmond, VA: BrainTrain. 1995.

90. Heaton RK. Revised comprehensive norms for an expanded Halstead-Reitan Battery: Demographically adjusted neuropsychological norms for African American

and Caucasian adults, professional manual: Psychological Assessment Resources; 2004.

91. Wechsler D. Manual for the Wechsler Adult Intelligence Scale. Oxford, England: Psychological Corp.; 1955. vi, 110-vi, p.

92. Borod JC, Goodglass H, Kaplan E. Normative data on the Boston diagnostic aphasia examination, parietal lobe battery, and the Boston naming test. Journal of

Clinical and Experimental Neuropsychology. 1980;2(3):209-15.

93. Erdodi LA, Tyson BT, Abeare CA, Lichtenstein JD, Pelletier CL, Rai JK, et al. The BDAE Complex Ideational Material—A measure of receptive language or

performance validity? Psychological Injury and Law. 2016;9:112-20.

94. Štěpánková H, Nikolai T, Lukavský J, Bezdíček O, Vrajová M, Kopeček M. Mini-mental state examination–česká normativní studie. Česká a slovenská neurologie a

neurochirurgie. 2015;78(111):1.

95. Bezdicek O, Motak L, Axelrod BN, Preiss M, Nikolai T, Vyhnalek M, et al. Czech version of the Trail Making Test: Normative data and clinical utility. Archives of

Clinical Neuropsychology. 2012;27(8):906-14.

96. Krivá Ľ. Stroopův test. Praha: Hogrefe–Testcentrum. 2013.

97. Wechsler D. WAIS-III-Wechslerova inteligenční škála pro dospělé. Praha: Hogrefe–Testcentrum. 2010.

98. Nikolai T, Stepankova H, Michalec J, Bezdicek O, Horakova K, Markova H, et al. Tests of verbal fluency, Czech normative study in older patients. CESKA A

SLOVENSKA NEUROLOGIE A NEUROCHIRURGIE. 2015;78(3):292-9.

99. Stepankova H, Lukavsky J, Buschkuehl M, Kopecek M, Ripova D, Jaeggi SM. The malleability of working memory and visuospatial skills: a randomized controlled

study in older adults. Developmental Psychology. 2014;50(4):1049.

100. Bezdicek O, Stepankova H, Moták L, Axelrod BN, Woodard JL, Preiss M, et al. Czech version of Rey Auditory Verbal Learning test: normative data. Aging,

Neuropsychology, and Cognition. 2014;21(6):693-721.

101. Preiss M, Rodriguez M, Laing H. Neuropsychologická baterie Psychiatrického centra Praha: Klinické vyšetření základních kognitivních funkcí: Psychiatrické

centrum; 2002.

102. Drozdova K, Stepankova H, Lukavsky J, Bezdicek O, Kopecek M. Normative data for the rey-osterrieth complex figure test in older Czech adults. Ceska a

Slovenska Neurologie a Neurochirurgie. 2015;78(5):542-9.

103. Mack WJ, Freed DM, Williams BW, Henderson VW. Boston Naming Test: shortened versions for use in Alzheimer's disease. Journal of gerontology.

1992;47(3):P154-P8.

104. Laingová H. The Visual Object and Space Perception Battery (Baterie testů vizuálního vnímání předmětů a prostoru). Hogrefe-Testcentrum, Praha. 2002.

105. Mitrushina M, Boone KB, Razani J, D'Elia LF. Handbook of normative data for neuropsychological assessment: Oxford University Press; 2005.

106. Conners CK, Staff M, Connelly V, Campbell S, MacLean M, Barnes J. Conners’ continuous performance Test II (CPT II v. 5). Multi-Health Syst Inc. 2000;29:175-

96.

107. Benedict RH, Schretlen D, Groninger L, Brandt J. Hopkins Verbal Learning Test–Revised: Normative data and analysis of inter-form and test-retest reliability. The

Clinical Neuropsychologist. 1998;12(1):43-55.

108. Benedict RH, Schretlen D, Groninger L, Dobraski M, Shpritz B. Revision of the Brief Visuospatial Memory Test: Studies of normal performance, reliability, and

validity. Psychological assessment. 1996;8(2):145.

**Supplementary Table 4. Neurocognitive test scores and between group comparisons.**

| **Authors, year** | **Test** | **Scores in FND** | **Scores in controls** | **Between groups comparisons** |
| --- | --- | --- | --- | --- |
| Almis et al., 2013 | WMS-R personal and actual findings | 4.77 (0.97) | 5.22 (0.75) | p=.090 |
|  | WMS-R orientation | 4.90 (0.52) | 5.00 (0.00) | p=.422 |
|  | WMS-R mental control | 6.86 (1.16) | 7.77 (1.10) | p=.023 |
|  | WMS-R logical memory | 7.47 (2.54) | 9.95 (1.81) | p=.001 |
|  | WMS-R logical memory long-term | 6.25 (2.32) | 13.40 (21.65) | p=.0001 |
|  | WMS-R forward number range | 4.40 (1.68) | 4.86 (1.72) | p=.381 |
|  | WMS-R reverse number range | 2.68 (1.55) | 3.72 (1.63) | p=.036 |
|  | WMS-R number range total | 7.09 (2.82) | 8.59 (2.97) | p=.094 |
|  | WMS-R visual recall instant | 11.13 (2.51) | 12.13 (1.12) | p=.096 |
|  | WMS-R visual recall long-term | 10.22 (2.77) | 11.77 (1.41) | p=.025 |
|  | Stroop 1 | 34.54 (8.11) | 27.18 (4.98) | p=.001 |
|  | Stroop 2 | 45.72 (13.72) | 34.50 (5.50) | p=.001 |
|  | Stroop 3 | 35.04 (8.72) | 27.40 (4.13) | p=.001 |
|  | Stroop 4 | 84.40 (25.56) | 69.31 (13.41) | p=.018 |
|  | Duration difference | 49.36 (19.70) | 41.90 (10.87) | p=.128 |
|  | Number of errors | 1.27 (1.45) | 0.50 (0.59) | p=.026 |
|  | Spontaneous correction | 3.63 (1.67) | 2.13 (1.64) | p=.005 |
| Ball et al., 2021  *For HVLT-R, age-corrected t scores were used in place of raw data | Trail Making Task-B (s) | 105 (74) | HC: 75 (38)  nMCI: 134 (82) | FCD vs HC: -.40 (0.02)  FCD vs nMCI: -0.02 (0.92) |
|  | HLVT-R Total recall (trials 1-3)* | 37.3 (12.3) | HC: 45.7 (10.6)  nMCI: 33.7 (8.8) | FCD vs HC: 9.13 (<.001)  FCD vs nMCI: -3.60 (0.31) |
|  | HLVT-R Delayed recall (trial 4)* | 40.3 (14.0) | HC: 49.2 (10.0)  nMCI: 31.8 (10.1) | FCD vs HC: 8.86 (0.02)  FCD vs nMCI: -8.52 (0.03) |
|  | HLVT-R Retention (trial 4/best of trial 2&3)* | 43.1 (14.1) | HC: 50.0 (9.9)  nMCI: 32.8 (12.1) | FCD vs HC: 6.99 (0.07)  FCD vs nMCI: -10.34 (0.01) |
|  | HLVT-R Recognition discrimination index (recognition hits minus false positives)* | 36.4 (14) | HC: 50.5 (8.3)  nMCI: 34.9 (11.5) | FCD vs HC: 14.12 (<.01)  FCD vs nMCI: -1.45 (0.71) |
|  | d-prime | 2.22 (1.58) | HC: 3.68 (0.84)  nMCI: 2.18 (1.12) | FCD vs HC: 12.62 (<.01)  FCD vs nMCI: -0.30 (0.94) |
| Bharambe & Larner, 2018 | Mini-Addenbrookes Cognitive Examination - ratio of individuals scoring above:below the cut-off | 31:12 (72.1% at or above cut-off)  n=43 | 30:6 (83.3% at or above cut-off)  n=36 | p>.10  Sensitivity, specificity and positive predictive values of MACE for diagnosis of FCD  Sensitivity: 0.72 (0.59-0.85)  Specificity: 0.17 (0.04-0.29)  PPV: 0.51 (0.38-0.63) |
| Bhome et al., 2019 | National Adult Reading Test | n=12  Superior: 3  Normal: 9  Inferior: 0 | FCD+depression n=6  Superior: 1  Normal: 5  Inferior: 0  FCD-depression n=6  Superior: 2  Normal: 4  Inferior: 0 | FCD+depression v FCD-depression  p=.51 |
|  | WAIS-III Verbal IQ | n=17  Superior: 4  Normal: 11  Inferior: 2 | FCD+depression n=10  Superior: 3  Normal: 5  Inferior: 2  FCD-depression n=7  Superior: 1  Normal: 6  Inferior: 0 | p=.27 |
|  | WAIS-III Non-verbal IQ | n=21  Superior: 3  Normal: 9  Inferior: 9 | FCD+depression n=12  Superior: 2  Normal: 5  Inferior: 5  FCD-depression n=9  Superior: 1  Normal: 4  Inferior: 4 | p=.94 |
|  | RMT Visual memory | n=21  Superior: 4  Normal: 8  Inferior: 9 | FCD+depression n=11  Superior: 3  Normal: 3  Inferior: 5  FCD-depression n=10  Superior: 1  Normal: 5  Inferior: 4 | p=.46 |
|  | RMT Verbal memory | n=22  Superior: 4  Normal: 7  Inferior: 11 | FCD+depression n=12  Superior: 2  Normal: 2  Inferior: 8  FCD-depression n=10  Superior: 2  Normal: 5  Inferior:3 | p=.18 |
|  | SDMT Processing speed | n=21  Superior: 3  Normal: 8  Inferior: 10 | FCD+depression n=12  Superior: 2  Normal: 4  Inferior: 6  FCD-depression n=9  Superior: 1  Normal: 4  Inferior:4 | p=.86 |
|  | VOSP Visuospatial | n=21  Normal: 18  Inferior: 3 | FCD+depression n=12  Normal: 9  Inferior: 3  FCD-depression n=9  Normal: 9  Inferior:0 | p=.16 |
| Binder et al., 1998 | WAIS-R Verbal IQ | 92.6 (13.1) | HC: 101.1 (15.1)  ES: 92.0 (15.9) | Kruskal-Wallis tests: comparisons of both seizure groups with HCs  Verbal IQ  χ2=11.35, p=.004 |
|  | WAIS-R Performance IQ | 93.3 (16.1) | HC: 105.0 (13.7)  ES: 90.8 (14.7) | χ2=20.46, p=.0001 |
|  | WAIS-R Full Scale IQ | 92.0 (14.3) | HC: 102.1 (15.4)  ES: 90.6 (15.3) | χ2=15.18, p=.001 |
|  | WCST Categories | 4.6 (1.8) | HC: 5.6 (1.1)  ES: 4.2 (2.1) | χ2=9.00, p=.012 |
|  | WCST Perseverative Responses | 26.0 (30.0) | HC: 15.9 (11.7)  ES: 31.3 (29.3) | χ2=6.62, p=.04 |
|  | Trail Making Task-A | 38.2 (18.4) | HC: 25.4 (9.4)  ES: 39.3 (26.4) | χ2=14.55, p=.001 |
|  | Trail Making Task-B | 93.0 (66.6) | HC: 62.0 (27.5)  ES: 97.2 (68.8) | χ2=14.51, p=.001 |
|  | Finger tapping dom | 45.4 (12.1) | HC: 53.2 (6.8)  ES: 46.0 (8.3) | χ2=19.56, p=.0001 |
|  | Finger tapping nondom | 40.5 (10.8) | HC: 49.0 (10.7)  ES: 40.4 (7.9) | χ2=25.84, p=.0001 |
|  | Grooved pegboard dom | 84.6 (24.3) | HC: 64.5 (7.3)  ES: 96.9 (34.0) | χ2=42.07, p=.0001 |
|  | Grooved pegboard nondom | 106.8 (72.9) | HC: 69.5 (11.4)  ES: 100.3 (33.8) | χ2=33.17, p=.006 |
|  | Finger agnosia errors | 4.3 (5.4) | HC: 0.7 (1.3)  ES: 2.7 (6.7) | χ2=9.08, p=.011 |
|  | Finger graphesthesia errors | 7.3 (6.1) | HC: 1.4 (1.6)  ES: 6.7 (6.0) | χ2=36.67, p=.0001 |
|  | WRAT-R Reading Standard Score | 94.8 (14.8) n=24 | ES: 92.1 (14.2) n=33 | t-tests: non-epileptic and epileptic group comparisons  t=.70, p=.49 |
|  | Boston Naming Test | 52.7 (6.8) n=23 | ES: 46.7 (9.4) n=38 | t=2.56, p=.02 |
|  | Controlled Oral Word Association Test | 34.0 (11.2) n=21 | ES: 32.1 (11.7) n=37 | t=.59, p=.56 |
|  | Digit Span Forward | 5.8 (1.2) n=27 | ES: 6.2 (1.4) n=38 | t=-1.24, p=.22 |
|  | Digit Span Backward | 4.7 (1.2) n=27 | ES: 4.4 (1.3) n=38 | t=.85, p=.40 |
|  | Rey AVLT Trial 1 | 5.8 (2.4) n=27 | ES: 5.5 (2.0) n=41 | t=.54, p=.60 |
|  | Rey AVLT Trial 5 | 11.2 (3.0) n=27 | ES: 10.4 (3.1) n=41 | t=1.09, p=.28 |
|  | Rey AVLT Trials 1-5, Total | 44.6 (12.0) n=27 | ES: 42.0 (12.1) n=41 | t=.85, p=.41 |
|  | Rey AVLT Brief Delayed Recall | 8.5 (3.8) n=27 | ES: 8.1 (3.8) n=41 | t=.42, p=.94 |
|  | Rey AVLT 20 Minute Delayed Recall | 8.3 (4.0) n=26 | ES: 7.6 (3.5) n=39 | t=.79, p=.94 |
|  | Rey AVLT Recognition, Right Minus Wrong | 9.6 (4.4) n=26 | ES: 9.1 (4.9) n=39 | t=.44, p=.67 |
|  | Rey Complex Figure Copy | 30.5 (6.8) n=24 | ES: 29.8 (6.4) n=38 | t=.41, p=.69 |
|  | Rey Complex Figure Delayed Recall | 16.9 (8.7) n=19 | ES: 16.5 (7.8) n=27 | t=.18, p=.86 |
|  | WMS-R Logical Memory I percentile | 30.1 (23.9) n=28 | ES: 34.4 (27.8) n=40 | t=-.66, p=.52 |
|  | WMS-R Logical Memory II percentile | 33.8 (24.6) n=28 | ES: 31.5 (26.3) n=40 | t=.35, p=.73 |
|  | WMS-R LM Savings percentage | 79.3 (16.6) n=28 | ES: 69.5 (25.8) n=40 | t=1.77, p=.09 |
|  | CVMT Total Correct | 72.0 (10.0) n=25 | ES: 72.4 (8.8) n=42 | t=-.20, p=.85 |
|  | Face Hand Errors | 4.7 (6.3) n=26 | ES: 2.4 (4.3) n=38 | t=1.73, p=.09 |
| Borreli et al., 2022 | Mini-Mental State Examinaton | 22.4 (6.2) | MCI: 21.4 (4.4)  ND: 13.6 (7.6) | Corrected p<.0001  Total MMSE scores was a predictor of FCD diagnosis p<.001, OR(95% CI)=0.84 (0.8-0.88) |
| Bortz et al., 1995 | CVLT  Response bias | -0.3533 (0.36) | LTF: +0.3083 (0.31)  RTF: +0.0700 (0.29) | (LTF > RTF > FS)  F=15.69, p=.0000 |
|  | CVLT False positives | 0.667 (0.77) | LTF: 3.750 (3.5)  RTF: 1.909 (1.9) | (LTF > FS)  F=7.34, p=.0020 |
|  | CVLT Intrusion errors | 2.944 (3.2) | LTF: 5.500 (4.9)  RTF: 4.090 (3.8) | (ns)  F=1.53, p=.2303 |
|  | CVLT Recognition hits | 12.27 (3.5) | LTF: 15.17 (1.0)  RTF: 14.45 (1.0) | (LTF > FS)  F=5.707, p=.0068 |
|  | CVLT Recognition hits vs. long-delay recall | 42.2 (14.8) | LTF: 67.50 (16.0)  RTF: 52.73 (12.7) | (LTF > FS)  F=10.72, p=.0002 |
| Brown et al., 1991 | WAIS-R Information | 8.8 (3.0) | 8.4 (3.2) | All nonsignificant (p- values not presented in manuscript) |
|  | WAIS-R Arithmetic | 9.0 (3.7) | 8.8 (3.2) |  |
|  | Boston Naming Test | 48.9 (8.8) | 45.3 (10.9) |  |
|  | Controlled Oral Word Association Test | 31.1 (12.4) | 30.4 (10.2) |  |
|  | WAIS-R Similarities | 8.0 (2.9) | 8.9 (2.5) |  |
|  | Proverb Interpretation  Multiple Choice | 16.4 (3.3) | 16.5 (3.9) |  |
|  | Proverb Interpretation  Spontaneous | 6.9 (2.4) | 5.8 (3.0) |  |
|  | WAIS-R Block Design | 7.6 (2.3) | 8.3 (2.3) |  |
|  | Judgment of Line Orientation | 7.1 (1.9) | 7.1 (2.1) |  |
|  | WAIS-R Digit Span | 7.5 (3.4) | 8.7 (3.5) |  |
|  | Benton Visual Retention Test | 13.8 (1.5) | 14.0 (1.5) |  |
|  | WMS Logical Memory Immediate | 11.3 (4.3) | 12.1 (4.4) |  |
|  | WMS Logical Memory Delayed | 10.2 (5.3) | 10.6 (4.7) |  |
|  | Paired Associates Easy | 5.6 (0.7) | 5.8 (0.5) |  |
|  | Paired Associates Hard | 3.2 (1.2) | 2.9 (1.3) |  |
| Brown et al., 2014 | National Adult Reading Test estimated IQ | 105.13 (10.19) | 108.75 (8.37) | t(54)=2.16, p=.04 |
|  | Trail Making Task-A: Time (s) | 35.96 (14.74) | 24.51 (8.98) | t(27)=-3.16, p=.004 |
|  | Trail Making Task-A: Errors | 0.40 (.50) | 0.28 (.51) | t(54)=-.86, p=.39 |
|  | Trail Making Task-B: Time (s) | 93.69 (66.83) | 55.47 (22.34) | t(21)=-2.48, p=.021 |
|  | Trail Making Task-B: Errors | 0.65 (2.46) | 0.53 (1.00) | t(54)=-.26, p=.79 |
|  | Time B-Time A (s) | 43.34 (34.16) | 26.63 (14.82) | t(22)=-2.13, p=.045 |
|  | Stroop test  Words: Time (s) | 85.68 (20.69) | 109.06 (15.75) |  |
|  | Stroop test  Colours: Time (s) | 64.68 (16.01) | 77.31 (12.24) |  |
|  | Stroop test  Inferference time (s) | 39.79 (11.81) | 47.83 (11.55) | t(53)=2.42, p=.019 |
|  | Logical memory  Immediate recall: Exact units | 37.76 (12.49) | 44.11 (7.81) | t(29)=2.10, p=.044 |
|  | Logical memory  Immediate recall: Thematic units | 16.14 (4.05) | 17.58 (2.84) | t(55)=1.57, p=.12 |
|  | Logical memory Delayed recall:  Exact units | 20.95 (10.35) | 28.42 (7.04) | t(31)=2.93, p=.006 |
|  | Logical memory Delayed recall:  Thematic units | 10.57 (2.96) | 11.75 (1.78) | t(29)=1.66, p=.11 |
|  | Auditory-verbal recognition | 25.14 (3.17) | 26.64 (2.03) | t(30)=1.94, p=.061 |
|  | Retention (%) | 74.06 (22.34) | 85.71 (11.93) | t(27)=2.21, p=.036 |
|  |  |  |  | *when adding estimated IQ as a covariate, many group differences were attenuated. The only remaining significant differences were:  TMT-A (s): p=.003  TMT-B (s): p=.026  Immediate recall exact units: p=.047  Delayed recall exact units: p=.02 |
| Caceres et al., 2021 | WAIS-III Vocabulary | 38.8 (8.5) n=23 | 39.2 (12.2) n=23 | Mann-Whitney U tests  p=.9 |
|  | WAIS-III Information | 13.1 (5.3) n=24 | 13.7 (6.6) n=23 | p=.73 |
|  | WAIS-III Block Design | 31.9 (13.6) n=18 | 37.2 (10.8) n=23 | p=.17 |
|  | RAVLT Trial 1 | 6.0 (1.8) n=24 | 6.0 (1.6) n=24 | p=.99 |
|  | WAIS-III Digit Span forward | 7.0 (2.0) n=24 | 6.8 (2.0) n=24 | p=.77 |
|  | TMT-A (s) | 41.8 (17.7) n=24 | 35.5 (18.1) n=24 | p=.23 |
|  | Grooved Pegboard Dominant (s) | 74.9 (14.3) n=23 | 69.7 (13.6) n=24 | p=0.2 |
|  | RAVLT Trial 5 | 12.5 (1.8) (n=24) | 12.0 (2.0) n=24 | p=0.37 |
|  | RAVLT Total Learning | 49.7 (8.3) n=24 | 47.6 (7.9) (n=24) | p=0.37 |
|  | RAVLT Delayed Memory | 10.4 (2.8) n=24 | 9.8 (2.8) n=24 | p=0.45 |
|  | Visual Memory (WMS-III): Faces Immediate | 38.5 (3.6) (n=22) | 37.4 (4.3) (n=24) | p=0.34 |
|  | Visual Memory (WMS-III): Faces % Retention | 98.1 (9.3) (n=22) | 97.7 (8.7) (n=24) | p=0.88 |
|  | Visual Memory (WMS-III): Visual Reproduction Immediate | 84.8 (15.4) (n=21) | 84.5 (12.1) (n=24) | p=.95 |
|  | Visual Memory (WMS-III): Visual Reproduction % Retention | 83.0 (13.6) (n=21) | 77.5 (20.6) (n=24) | p=.3 |
|  | Boston Naming Test | 52 (4.3) (n=23) | 50.0 (6.3) (n=24) | p=.21 |
|  | Semantic verbal fluency (animals) | 21.3 (6.2) (n=24) | 19.4 (4.7) (n=24) | p=.24 |
|  | Trail Making Task-B (s) | 119.1 (91.5) (n=24) | 98.0 (86.0) (n=24) | p=.41 |
|  | WAIS-III Digit Span backward | 5.1 (1.7) n=24 | 5.5 (1.8) n=24 | p=.5 |
|  | Phonemic Verbal Fluency (total 3 letters) | 36.8 (14.7) n=23 | 37.1 (12.8) n=24 | p=.04 |
| Criswell et al., 2010  *scores adjusted for age | Combined Finger Tapping Test | 50.42 (15.89)  41.72* | HC: 74.31 (11.61)  Dystonia: 68.37 (12.30)  Essential tremor: 61.23 (16.42)  IPD: 56.79 (13.55)  HC: 74.31*  Dystonia: 66.31*  Essential tremor: 60.10*  IPD: 58.78* | Combined mean FTT between diagnostic categories: F=31.72, p<0.001  Combined mean FTT between diagnostic categories when controlling for age: F = 36.37, p < 0.001  Tukey's HSD post-hoc comparison: FMS group performed fewer taps than the IPD, ET, dystonia, and HC.  FMS vs IPD: difference of 17.06 taps (p<0.001) |
| de Vroege et al., 2021 | WAIS-IV Digit Symbol Substitution Test | 57.2 (19.1) n=26  No problems: 9 (34.6%)  Deficit: 11 (42.3%)  Disorder: 6 (23.1%) | 64.2 (17.6) n=251 | d=−.39 |
|  | Trail Making Task-A (s) | 43.2 (17.5) n=27  No problems: 12 (44.4%)  Deficit: 10 (37%)  Disorder: 5 (18.5%) | 37.3 (18.9) n=259 | U=2,579.5, z=−2.243, p=.025, d=−.31 |
|  | Stroop Color-Word Test Card 1 | 62.0 (15.1) n=26  No problems: 3 (11.5%)  Deficit: 10 (38.5%)  Disorder: 13 (50%) | 50.6 (13.8) n=259 | U=1,816.5, z=−3.872, p<.001, d=−.82 |
|  | Stroop Color-Word Test Card 2 | 79.6 (24.6) n=25  No problems: 6 (24.0%)  Deficit: 4 (16%)  Disorder: 15 (60%) | 63.9 (17.1) n=259 | U=1,928.5, z=−3.339, p=.001, d=−.88 |
|  | Trail Making Task-B | 104.6 (60.2) n=25  No problems: 16 (64.0%)  Deficit: 4 (16%)  Disorder: 5 (20%) | 82.5 (47.6) n=257 | d=−.45 |
|  | Stroop Color-Word Test Card 3 | 124.8 (61.8) n=25  No problems: 22 (88.0%)  Deficit: 3 (12%)  Disorder: 0 | 102.4 (39.0) n=258 | U=2,375.5, z = −2.175, p=.030, d=−.54 |
|  | d2 | 132.5 (46.1) n=23  No problems: 11 (47.8%)  Deficit: 10 (43.5%)  Disorder: 2 (8.7%) | 144.3 (46.1) n=250 | d=.26 |
|  | RAVLT immediate recall | 40.2 (11.7) n=27  No problems: 17 (63.0%)  Deficit: 3 (11.1%)  Disorder: 7 (25.9%) | 42.2 (10.8) n=261 | d=.18 |
|  | RBMT Story immediate recall | 16.4 (6.1) n=26  No problems: 20 (76.9%)  Deficit: 6 (23.1%)  Disorder: 0 | 17.1 (6.1) n=260 | d=.11 |
|  | RAVLT delayed recall | 8.1 (3.7) n=27  No problems: 16 (59.3%)  Deficit: 6 (22.2%)  Disorder: 5 (18.5%) | 8.6 (3.1) n=262 | d=.06 |
|  | RBMT Story delayed recall | 12.7 (6.0) n=26  No problems: 19 (73.1%)  Deficit: 5 (19.2%)  Disorder: 2 (7.7%) | 13.9 (5.8) n=256 | d=.21 |
|  | ROCFT immediate recall | 17.9 (7.7) n=26  No problems: 13 (50%)  Deficit: 9 (34.6%)  Disorder: 4 (15.4%) | 18.9 (7.0) n=253 | d=.14 |
|  | ROCFT delayed recall | 16.0 (7.3) n=26  No problems: 12 (46.2%)  Deficit: 6 (23.1%)  Disorder: 8 (30.8%) | 18.5 (6.8) n=252 | d=.37 |
|  | Boston Naming Test | 159.0 (13.4) n=25  No problems: 15 (60.0%)  Deficit: 7 (28.0%)  Disorder: 3 (12.0%) | 157.8 (16.3) n=260 | d=−.07 |
|  | ROCFT copy | 28.6 (6.4) n=26  No problems: 0  Deficit: 18 (69.2%)  Disorder: 8 (30.8%) | 31.0 (5.3) n=256 | U=2,527.0, z=−2.031, p=.042, d=.44 |
|  | Phonological verbal fluency: N + A | 18.2 (7.8) n=25  No problems: 12 (52.2%)  Deficit: 11 (47.8%)  Disorder: 0 | 22.3 (9.2) n=252 | U=2,382.5, z=−2.010, p=.044, d=.45 |
|  | Semantic verbal fluency: animal naming | 30.2 (8.1) n=25  No problems: 21 (84.0%)  Deficit: 3 (12.0%)  Disorder: 1 (4.0%) | 32.4 (8.9) n=252 | d=.25 |
|  | WAIS-IV Digit Span | 22.2 (5.9) n=27  No problems: 13 (48.1%)  Deficit: 8 (29.6%)  Disorder: 6 (22.2%) | 24.4 (5.2) n=261 | d=.42 |
|  | BADS Rule Shift Cards | 18.7 (2.7) n=26 | 19.0 (2.4) n=252 | d=.12 |
|  | BADS Key Search | 11.9 (4.3) n=27  No problems: 21 (77.8%)  Deficit: 1 (3.7%)  Disorder: 5 (18.5%) | 11.9 (3.8) n=255 | d=.00 |
|  | BADS Zoo Map | 11.5 (4.5) n=26  No problems: 24 (92.3%)  Deficit: 2 (7.7%)  Disorder: 0 | 11.6 (4.0) n=250 | d=.02 |
| Demartini et al., 2014 | Reading the Mind in the Eyes Test | 23.38 (4.3) | OMD: 22.73 (4.1)  HC: 24.21 (3.9) | p=.353 |
| Demartini et al., 2019 | Mini-Mental State Examination | 29.60 (0.96) | 29.90 (0.31) | t=-9.33, p=.363 |
| Demir et al., 2013 | Serial Digit Learning Test | 9.10 (8.22)  Unable to learn (n %) 20 (46.5)  Learners (n %)  23 (53.5) | PD: 15.30 (6.54)  HC: 14.56 (7.22)  Unable to learn (n %)  PD: 5 (11.4)  HC: 7 (16.3)  Learners (n %)  PD: 39 (88.6)  HC: 36 (83.7) | P1 = FND & HC  P2= FND & PD  P1: p=.004  P2: p<.001 |
|  | AVLT Maximal learning | 12.84 (1.95) | PD: 14.30 (1.25)  HC: 14.14 (1.75) | P1: p<.001  P2: p<.001 |
|  | AVLT delayed memory | 10.51 (2.41) | PD: 12.80 (1.66)  HC: 12.77 (2.16) | P1: p<.001  P2: p<.001 |
|  | AVLT Total learning | 101.98 (18.17)  Unable to learn (n %)  34 (79.1)  Learners (n %)  9 (20.9) | PD: 115.09 (15.76)  HC: 117.07 (19.68)  Unable to learn (n %)  PD: 15 (34.1)  HC: 29 (65.9)  Learners (n %)  PD: 12 (27.9)  HC: 31 (72.1) | P1: p<.001  P2: p<.002 |
|  | WMS general information | 5.81 (0.45) | PD: 5.89 (0.39)  HC: 6.00 (0.00 | P1: p=.006  P2: p=.327 |
|  | WMS orientation | 4.77 (0.43) | PD: 4.93 (0.25)  HC: 4.98 (0.15) | P1: p=.004  P2: p=.033 |
|  | WMS mental control | 6.63 (1.63) | PD: 8.41 (1.04)  HC: 8.58 (0.76) | P1: p<.001  P2: p<.001 |
|  | WMS logical memory | 5.36 (3.03) | PD: 7.20 (2.61)  HC: 7.24 (2.18) | P1: p=.003  P2: p=.004 |
|  | Stroop 1 score | 11.49 (3.43) | PD: 8.68 (2.37)  HC: 8.81 (1.96) | P1: p<.001  P2: p<.001 |
|  | Stroop 2 score | 12.38 (3.67) | PD: 9.76 (3.94)  HC: 9.87 (3.02) | P1: p=.004  P2: p=.002 |
|  | Stroop 3 score | 15.19 (4.29) | PD: 12.39 (2.24)  HC: 12.87 (3.80) | P1: p=.005  P2: p=.001 |
|  | Stroop 4 score | 21.93 (7.88) | PD: 18.03 (4.01)  HC: 18.54 (6.31) | P1: p=.039  P2: p=.030 |
|  | Stroop 5 score | 32.41 (11.46) | PD: 24.49 (5.71)  HC: 27.16 (9.43) | P1: p=.029  P2: p=.001 |
|  | BJLOT correct response (n) | 17.56 (4.74) | PD: 20.32 (4.64)  HC: 21.19 (4.28) | P1: p=.001  P2: p=.015 |
|  | CT time to complete structured letter test (min) | 128.23 (42.65) | PD: 98.55 (21.93)  HC: 106.91 (29.16) | P1: p=.014  P2: p<.001 |
|  | CT time to complete structured shape test (min) | 106.14 (31.21) | PD: 86.50 (16.31)  HC: 93.65 (25.37) | P1: p=.054  P2: p=.001 |
|  | CT time to complete random letters test (min) | 121.60 (38.83) | PD: 100.27 (16.10)  HC: 107.35 (31.21) | P1: p=.111  P2: p=.013 |
|  | CT time to complete random shapes test (min) | 96.58 (31.81) | PD: 79.86 (15.02)  HC: 84.51 (25.62) | P1: p=.080  P2: p=.008 |
|  | CT random shapes total # of errors | 3.91 (3.29) | PD: 3.16 (2.23)  HC: 2.51 (2.19) | P1: p=.039  P2: p=.380 |
| Drane et al., 2006 | Dodrill Discrimination Index (SD)  Percentage of tests on which a patient scores below the normal limits | 52.6 (27.7) n=37 | 50.3 (25.0) n=37 | Statistics not presented |
| Dunbar et al., 2021  *analysed standardized T scores for each measure | CPT-III Response Style* | 50.1 (11.6) | 52.4 (13.7) | p=.388 |
|  | CPT-III Detectability* | 58.6 (11.1) | 52.8 (11.7) | p=.054 |
|  | CPT-III Omissions* | 58.1 (14.5) | 53.9 (13.5) | p=.226 |
|  | CPT-III Commissions* | 57.1 (12.2) | 51.4 (12.4) | p=.037 |
|  | CPT-III Perseveration* | 53.6 (11.8) | 52.9 (12.0) | p=.970 |
|  | CPT-III Hit reaction time (HRT)* | 57.3 (11.1) | 60.4 (11.6) | p=.301 |
|  | CPT-III HRT standard deviation* | 57.1 (15.0) | 49.3 (10.9) | p=.036 |
|  | Variability* | 51.2 (12.4) | 51.0 (12.4) | p=.354 |
|  | CPT-III HRT block change* | 50.6 (12.4) | 51.0 (9.9) | p=.921 |
|  |  | % CPT+ = 54.5 | % CPT+ = 53.7 | %CPT+  p>.999 |
| Giugno et al., 2023 | Controlled Oral Word Association Test | **Median (IQR)**  34.00 (14.075) | **Median (IQR)**  TLE: 34.50 (16.00) | Post hoc Bonferroni tests  TLE vs FS p=.338 |
|  | Digit Span Forward | 6.00 (3.00) | TLE: 6.00 (1.00) | TLE vs FS p=1.00 |
|  | Digit Span Backward | 4.00 (2.00) | TLE: 4.00 (0.25) | TLE vs FS p=1.00 |
|  | RAVLT | 38.00 (7.75) | TLE: 35.00 (10.25) | TLE vs FS p=1.00 |
|  | RAVLT-D | 7.00 (3.00) | TLE: 7.00 (2.25) | TLE vs FS p=1.00 |
|  | ROCFT | 25.00 (12.02) | TLE: 34.00 (6.13) | TLE vs FS p=.265 |
|  | ROCFT-D | 10.77 (5.38) | TLE: 10.10 (8.25) | TLE vs FS p=1.00 |
|  | Weigl Color-Form Sorting Test | 12.00 (2.96) | TLE: 10.00 (0.070) | TLE vs FS p=.069 |
| Gursoy et al., 2021 | Reading the Mind in the Eyes Test | 20.71 (4.15) | ES: 21.75 (3.74)  HC: 23.86 (2.66) | p=.005  FS group had significantly lower scores than HC group  No difference between FS and ES |
| Hamouda et al., 2021 | WAIS-IV Digit Span Forward | 8.67 (2.71) | 10.03 (1.74) | t(58.78)=−2.56, p=.01, d=0.62 |
|  | WAIS-IV Digit Span Backward | 6.13 (1.71) | 7.26 (2.44) | t(68.50)=−2.23, p=.03, d = 0.62 |
|  | Trail Making Task Difference Score | 27.74 (16.66) n=38 | 19.84 (9.62) n=38 | t(55.38)=2.45, p=.02, d=.67 |
|  | ANT Alerting (ms) | 46.54 (29.81) n=36 | 42.99 (24.38) n=39 | t(67.74)=.56, p=.58, d=0.013 |
|  | ANT Orienting (ms) | 50.93 (27.59) n=36 | 39.95 (26.50) n=39 | t(71.94)=1.76, p=.08, d = 0.41 |
|  | ANT Executive (ms) | 120.81 (45.01) n=36 | 113.35 (30.50) n=39 | t(59.01)=0.82, p=.42, d=0.19 |
| Heintz et al., 2013  *Used T scores (mean=50, SD=10) corrected for age (category fluency) or age and education (other tests) | Trail Making Task-A* | 52.4 (8.8) | GTS: 52.1 (9.7)  HC: 55.7 (11.4) | F=.88, p=.42 |
|  | Stroop Card I (word)* | 50.8 (8.0) | GTS: 49.1 (13.5)  HC: 51.4 (9.7) | F=.26, p=.77 |
|  | Stroop Card II (colour)* | 51.9 (9.2) | GTS: 45.1 (10.0)  HC: 55.0 (9.0) | F=5.21, p<.01  *Lower T scores in GTS than HC |
|  | Trail Making Task-B* | 49.9 (7.9) | GTS: 50.9 (9.9)  HC: 54.7 (11.8) | F=1.02, p=.22 |
|  | Stroop Card III (word colour)* | 52.9 (6.4) | GTS: 49.1 (11.3)  HC: 54.7 (8.5) | F=1.22, p=.13 |
|  | Semantic fluency* | 48.5 (8.2) | GTS: 45.4 (7.9)  HC: 49.1 (8.3) | F=1.57, p=.37 |
|  | Controlled Oral Word Association Test* | 47.0 (11.7) | GTS: 49.4 (10.3)  HC: 51.7 (8.2) | F=2.14, p=.30 |
|  | AVLT immediate recall* | 40.7 (8.8) | GTS: 48.9 (9.9)  HC: 49.2 (10.1) | F=6.02, p<.01  *FMS group performed ~1SD below the expected level |
|  | AVLT delayed recall* | 47.5 (19.9) | GTS: 52.8 (8.1)  HC: 52.9 (10.4) | F=2.17, p=.12 |
|  | RBMT immediate recall* | 49.4 (9.9) | GTS: 45.9 (11.2)  HC: 49.3 (10.6 | F=.52, p=.60 |
|  | RBMT delayed recall* | 47.4 (10.8) | GTS: 44.8 (13.1)  HC: 49.6 (8.9) | F=.88, p=.42 |
|  | WMS immediate recall* | 46.6 (6.9) | GTS: 44.7 (9.7)  HC: 47.1 (10.2) | F=.37, p=.70 |
|  | WMS delayed recall* | 48.2 (12.1) | GTS: 51.1 (8.3)  HC: 50.6 (9.2) | F=.48, p=.62 |
|  | Vienna Test System reaction times S1* | 53.4 (10.1) | GTS: 55.1 (5.5)  HC: 57.8 (7.1) | F=1.72, p=.19 |
|  | Vienna Test System reaction times S3* | 44.1 (9.7) | GTS: 46.9 (6.0)  HC: 48.7 (9.1) | F=1.17, p=.19 |
| Hill et al., 2003  *Percentile score | Logical Memory: Immediate* | 38.23 (28.66) | 30.67 (26.18) | p=.18 |
| ^Scaled score (M=100, SD=15) | Logical Memory: Delayed* | 32.66 (26.88) | 21.85 (20.69) | p=.03 |
| ~Scaled score (M=10, SD=3) | Visual Reproduction: Immediate* | 35.08 (28.72) | 40.98 (26.01) | p=.29 |
| +T-score | Visual Reproduction: Delayed* | 29.75 (26.58) | 23.48 (20.58) | p=.20 |
| “Raw score | Verbal Memory Index^ | 89.24 (18.00) | 84.79 (15.02) | p=.18 |
|  | Visual Memory Index^ | 90.26 (16.34) | 90.32 (15.75) | p=.99 |
|  | General Memory Index^ | 88.11 (18.69) | 83.91 (14.79) | p=.22 |
|  | Delayed Memory Index^ | 87.28 (17.95) | 77.81 (13.79) | p<.01 |
|  | Information~ | 7.98 (2.60) | 7.47 (2.42) | p=.31 |
|  | Vocabulary~ | 8.00 (2.61) | 8.94 (2.88) | p=.11 |
|  | Digit Span~ | 7.36 (2.28) | 8.50 (2.62) | p=.09 |
|  | Arithmetic~ | 7.87 (2.22) | 8.70 (2.39) | p=.07 |
|  | Comprehension~ | 7.79 (2.48) | 8.34 (3.14) | p=.36 |
|  | Similarities~ | 8.63 (2.30) | 9.26 (2.72) | p=.27 |
|  | Picture Completion~ | 8.94 (2.76) | 8.66 (2.55) | p=.59 |
|  | Picture Arrangement~ | 8.48 (2.37) | 9.15 (3.00) | p=.26 |
|  | Block Design~ | 8.61 (2.76) | 9.47 (2.58) | p=.11 |
|  | Object Assembly~ | 7.75 (1.93) | 7.87 (2.49) | p=.78 |
|  | Digit Symbol~ | 8.67 (2.43) | 8.85 (2.64) | p=.72 |
|  | Verbal IQ^ | 90.58 (13.34) | 88.60 (9.73) | p=.40 |
|  | Performance IQ^ | 90.76 (12.62) | 90.66 (10.98) | p=.97 |
|  | Full Scale IQ^ | 89.83 (11.98) | 88.49 (9.00) | p=.53 |
|  | CVLT Total+ | 34.94 (18.89) | 30.19 (16.84) | p=.19 |
|  | CVLT Trial 5” | 11.46 (3.32) | 10.60 (3.07) | p=.18 |
|  | CVLT Short Delay” | 9.63 (3.96) | 8.47 (3.60) | p=.13 |
|  | CVLT Long Delay” | 9.70 (4.09) | 8.53 (4.05) | p=.15 |
|  | CVLT Recognition” | 13.87 (2.46) | 13.51 (2.57) | p=.47 |
|  | CVLT False Positives” | 1.72 (2.41) | 2.85 (3.01) | p=.04 |
|  | CVLT Discrimination” | 91.25 (8.78) | 87.86 (8.60) | p=.05 |
| Hill & Gale, 2011  ^Index score | WASI Full Scale IQ^ | FS-NM: 96.0 (14.1)  FS-M: 89.0 (14.9) | 91.8 (14.7) | FS-NM vs FS-M: p<.01 FS-NM vs TLE: p<.05 |
|  | WASI Verbal IQ^ | FS-NM: 94.7 (14.1)  FS-M: 88.9 (14.7) | 89.1 (14.3) | FS-NM vs FS-M: p<.05 FS-NM vs TLE: p<.01 |
|  | WASI Performance IQ^ | FS-NM: 97.3 (14.2)  FS-M: 91.3 (15.1) | 96.1 (15.5) | FS-NM vs FS-M: p<.05 FS-M vs TLE: p<.05 |
|  | WAIS-III Digit Span scaled score | FS-NM: 9.4 (3.3)  FS-M: 8.4 (3.1) | 8.5 (2.8) | FS-NM vs TLE: p<.05 |
| *T score | Trail Making Task-A* | FS-NM: 45.3 (12.5)  FS-M: 43.3 (10.0) | 43.0 (12.2) | ns |
|  | Trail Making Task-B* | FS-NM: 44.0 (12.3)  FS-M: 44.2 (10.8) | 41.7 (11.5) | ns |
|  | Boston Naming Test | FS-NM: 53.3 (4.6)  FS-M: 51.6 (6.4) | 46.4 (9.5) | FS-NM vs TLE: p<.01  FS-M vs TLE: p<.01 |
|  | BVMT-R  Trials 1-3 Total* | FS-NM: 40.0 (13.6)  FS-M: 36.5 (13.1) | 36.3 (13.4) | FS-NM vs TLE: p<.05  FS-M vs TLE: ns |
|  | BVMT-R  Long Delay* | FS-NM: 42.0 (15.0)  FS-M: 37.5 (15.4) | 37.6 (14.1) | FS-NM vs TLE: p<.05  FS-M vs TLE: ns |
|  | RAVLT  Trials 1-5 Total* | FS-NM: 46.0 (14.1)  FS-M: 41.8 (13.4) | 40.3 (12.7) | FS-NM vs TLE: p<.01  FS-NM vs FS-M: p<.05 |
|  | RAVLT  Long Delay* | FS-NM: 46.8 (15.2)  FS-M: 43.0 (13.4) | 40.4 (12.4) | FS-NM vs TLE: p<.01  FS-M vs TLE: ns |
| Huys et al., 2020 | Cue RT in ms: None | 754.6 (152.0) | OMD: 674.9 (99.8)  HC: 638.9 (94.6) |  |
|  | Cue RT in ms: Center | 738.7 (144.7) | OMD: 651.2 (99.8)  HC: 612.3 (99.0) |  |
|  | Cue RT in ms: Double | 721.4 (137.8) | OMD: 636.8 (97.7)  HC: 602.6 (92.4) |  |
|  | Cue RT in ms: Spatial | 684.9 (145.2) | OMD: 595.2 (96.3)  HC: 566.0 (97.5) |  |
|  | Flanker RT in ms: Congruent | 685.0 (139.7) | OMD: 606.8 (94.5)  HC: 576.4 (90.7) |  |
|  | Flanker RT in ms: Neutral | 683.3 (136.4) | OMD: 605.2 (92.2)  HC: 575.9 (92.0) |  |
|  | Flanker RT in ms: Incongruent | 812.3 (158.0) | OMD: 709.8 (109.4)  HC: 666.3 (106.0) |  |
|  | Overall RT in ms | 724.7 (144.0) | OMD: 639.3 (97.4)  HC: 604.7 (95.0) | F(2,87)=8.74, p=.0003, pes=.17  post-hoc two-sample t-tests with Šidák-Holm correction:  FMS vs. HC: t_uncorr_(58)=−3.81, p_corr_=0.001, d=−0.98  FMS vs. OC: t_uncorr_(58)=−2.69, p_corr_=0.019, d=−0.69  HC vs. OC t_uncorr_(58)=−1.39, p_corr_=0.17, d=−0.36 |
|  | Alerting | 33.2, (26.8) | OMD: 38.1 (24.5)  HC: 36.3 (17.4) | F(2,87)=0.34, p=0.71, η^2^=0.008 |
|  | Orienting | 53.8 (27.9) | OMD: 56.0 (28.3)  HC: 46.4 (28.0) | F(2,87)=0.96, p=0.39, η^2^=0.022 |
|  | Conflict | 127.3 (38.9) | OMD: 103.1 (35.8)  HC: 90.0 (28.0) | F(2,87)=9.03, p=0.0003, η^2^=0.17  FMS vs. HC two-sample t-tests  t_uncorr_(58)=−4.27, p_corr_<0.0002, d=−1.1  FMS vs. OMD Wilcoxon rank sum test: z_uncorr_=−2.53, p_corr_=0.023, r=−0.33  HC vs. OMD Wilcoxon rank sum test: z_uncorr_=−1.21, p_corr_=0.23, r=−0.16 |
| Irorutola et al., 2020 | Reading the Mind in the Eyes Test | 24.54 (4.33) | 25.40 (3.77) | p=.15, d=.32 |
| Kramska et al., 2022 | List Learning | 27.68 (5.767)  EI<3: 28.4 (5.1)  EI>3: 21.4 (7.0) |  | Comparison of EI<3 vs EI>3 subgroups  p<.001 |
|  | Story Memory | 16.32 (4.213)  EI<3: 16.8 (3.9)  EI>3: 12.2 (4.8) |  | p<.001 |
|  | Figure Copy | 17.23 (2.616)  EI<3: 17.4 (2.6)  EI>3: 16.0 (2.3) |  | p=.014 |
|  | Line Orientation | 18.00 (6.824)  EI<3: 17.8 (2.8)  EI>3: 15.9 (3.6) |  | p=.021 |
|  | Picture Naming | 9.82 (0.617)  EI<3: 9.9 (0.5)  EI>3: 9.5 (1.3) |  | p=.183 |
|  | Semantic Fluency | 20.81 (5.921)  EI<3: 21.3 (5.7)  EI>3: 16.6 (5.7) |  | p=.001 |
|  | Digit Span | 8.68 (2.189)  EI<3: 9.0 (2.0)  EI>3: 5.6 (1.5) |  | p<.001 |
|  | Coding | 38.95 (13.207)  EI<3: 40.2 (12.9)  EI>3: 27.8 (10.6) |  | p<.001 |
|  | List Recall | 5.94 (2.384)  EI<3: 6.3 (2.1)  EI>3: 2.9 (2.4) |  | p<.001 |
|  | List Recognition | 18.76 (1.955)  EI<3: 19.2 (1.3)  EI>3: 15.1 (3.0) |  | p<.001 |
|  | Story Recall | 9.14 (2.347)  EI<3: 9.4 (2.2)  EI>3: 6.6 (2.4) |  | p<.001 |
|  | Figure Recall | 14.18 (4.013)  EI<3: 14.4 (3.8)  EI>3: 12.0 (4.7) |  | p=.019 |
|  | Immediate Memory | 90.89 (17.871)  EI<3: 92.8 (16.2)  EI>3: 73.5 (21.8) |  | p<.001 |
|  | Visuospatial/constructional | 94.54 (17.174)  EI<3: 95.6 (16.9)  EI>3: 85.4 (16.5) |  | p=.008 |
|  | Language | 97.39 (14.975)  EI<3: 98.6 (14.2)  EI>3: 86.7 (16.8) |  | p=.002 |
|  | Attention | 74.27 (17.591)  EI<3: 76.7 (16.4)  EI>3: 52.8 (12.4) |  | p<.001 |
|  | Delayed Recall | 90.06 (17.888)  EI<3: 92.7 (15.6)  EI>3: 65.7 (18.7) |  | p<.001 |
|  | Total Scale | 86.22 (15.757)  EI<3: 88.4 (14.4)  EI>3: 66.6 (13.9) |  | p<.001 |
| Leon-Sarmiento et al., 2019 | Picture Identification Test | 38 (0.06) | HC: 39.8 (0.10)  PD: 39.8 (0.33) | p=.10 |
| Lloyd et al., 2002 | Neuropsychiatry Unit Cognitive Assessment Tool | 88.68 (8.79) | 84.91 (12.20) | g=.32 [0.02, 0.63] |
| Matthews et al., 2020 | Montreal Cognitive Assessment | 26 (2.5) | HC: 28 (1.6)  OMD: 26 (2.8) | BF_10_=14.9, F(2,57)=6.6, p=.003  FMS: Difference (BF_10_ > 5 & Holm-corrected post-hoc comparison p<.05) vs. Control  OMD: Difference (BF_10_>5 & Holm-corrected post-hoc comparison p<.05) vs. Control  HC: Difference (BF_10_ > 5 & Holm-corrected post-hoc comparison p<.05) vs. FMS |
| McWhirter et al., 2021 | Clinical Addenbrookes Cognitive Examination iii | 87.9 (10) | 84 (7.72) |  |
|  | Montreal Cognitive Assessment total score | 21.9 (4.6) | 20.3 (3.1) | Univariate p (Holm-Bonferroni)  p=1 |
|  | Montreal Cognitive Assessment total time | 467 (82) | 506 (84) | p=1 |
|  | Montreal Cognitive Assessment orientation | 5.6 (0.8) | 4.8 (1.6) | p=1 |
| Myers et al., 2014 | WASI FSIQ | 95.65 (12.971)* n=17 | FS+PTSD: 89.41 (10.90)*  n=17  FS+trauma: 92.22 (13.694)*  n=29 | F=1, p=.372 |
|  | CVLT-II DR | 8.41 (3.337) n=17 | FS+PTSD: 7.87 (4.26) n=15  FS+trauma: 9.59 (3.627) n=29 | F=1.29, p=.283 |
|  | CVMT DR | 4 (1.541) n=17 | FS+PTSD: 3.38 (2.06) n=16  FS+trauma: 3.75 (1.951) n=29 | F=.461, p=.633 |
|  | D-KEFS T4 | 7.12 (4.136) n=17 | FS+PTSD: 7.13 (2.92) n=15  FS+trauma: 8.97 (2.927) n=29 | F=2.47, p=.093 |
|  | D-KEFS CW3 | 7.75 (3.568) n=16 | FS+PTSD: 8.46 (3.23) n=13  FS+trauma: 6.94 (3.549) n=29 | F=.94, p=.397 |
|  | Boston Naming Test | 47.53 (8.931) n=17 | FS+PTSD: 50.73 (4.03) n=15  FS+trauma: 48.45 (10.049) n=29 | F=.58, p=.565 |
|  | WMS Logical Memory I | 10.47 (2.375) n=17 | FS+PTSD: 9.13 (2.527) n=16  FS+trauma: 9.19 (2.429) n=29 | F=1.8, p=.174 |
|  | WMS Logical Memory II | 10.82 (1.944) n=17 | FS+PTSD: 8.69 (2.243) n=16  FS+trauma: 9.72 (2.439) n=29 | F=3.65, p=.032*  *FS+PTSD group had lower scores compared to FS+trauma and PNES alone, with significantly lower scores compared to the FS alone group (p=.024) |
| O’Brien et al., 2015 | WASI FSIQ | 103.1 (10.8) | 122.1 (9.9) | Logistic regression analysis  p<.001 |
|  | WTAR Standard IQ | 91.0 (14.0) | 115.4 (11.0) | p<.001 |
|  | SWM Between errors | 23.1 (16.9) | 18.8 (17.7) | *p-values adjusted for FSIQ  p=.453  *p=.025 |
|  | SWM between errors (6 boxes) | 6.2 (6.0) | 4.9 (6.6) | p=.542  *p=.042 |
|  | SWM between errors (8 boxes) | 16.3 (12.3) | 13.5 (11.9) | p=.482  *p=.036 |
|  | SWM total errors | 23.6 (17.3) | 19.9 (17.3) | p=.510  *p=.025 |
|  | SWM total errors (6 boxes) | 6.3 (6.1) | 5.3 (6.6) | p=.632  *p=.041 |
|  | SWM total errors (8 boxes) | 16.7 (12.7) | 14.2 (11.6) | p=.525  *p=.035 |
|  | SWM strategy | 30.2 (7.0) | 28.3 (7.1) | p=.423  *p=.048 |
|  | SOC mean initial thinking time (3 moves) | 4854 (2651) | 6264 (4945) | p=.281  *p=.031 |
|  | Rapid Visual Processing mean latency | 405.1 (55.8) | 407.5 (70.9) | p=.911  *p=.040 |
| O’Malley et al., 2020 | Mini-Mental State Examination | 27.3 (1.9) | AD: 23 (3.3)  MCI: 27.0 (0.8) | ns |
|  | Addenbrooke’s Cognitive Examination-Revised | 88.7 (5.9) | AD: 69.5 (8.2)  MCI: 81.3 (3.6)  HC: 95.3 (1.6) | HC vs FCD: p>.05  HC vs MCI: p<.05  HC vs AD: p<.05  FCD vs MCI: p<.05  FCD vs AD: p<.05  MCI vs AD: p<.05 |
| Ozer Celik et al., 2015 | VMPT immediate learning score | 5.70 (1.92) | ES: 6.27 (1.61)  HC: 6.50 (2.64) | Stats not presented |
|  | VMPT learning score | 104.45 (25.39) | ES: 101.18 (22.32)  HC: 122.35 (14.74) | FSvHC: p=.023  EvsHC: p=.012 |
|  | VMPT long-term memory | 11.85 (3.45) | ES: 10.36 (2.97)  HC: 13.10 (1.77) | EvsHC: p=.003 |
|  | VMPT recognition | 3.30 (3.64) | ES: 4.18 (2.52)  HC: 1.85 (1.75) | Stats not presented |
|  | Total recognition | 14.45 (4.75) | ES: 14.54 (0.68)  HC: 15.00 (0.00) | EvsHC: p=.005 |
| Pennington et al., 2015 | Montreal Cognitive Assessment (n=18) | 7 (39%) in normal range – median score of those in normal range = 29/30  11 patients who scored below the cut off, median score = 21/30 |  |  |
| Pennington et al., 2019 | Montreal Cognitive Assessment | 23.9 (no SD) | MCI: 23.3 (no SD)  HC: 27.8 (no SD) | Kruskal-Wallis test  Chi-square=19.0, df=2, p<.01  FCD vs HC  p<.01  FCD vs MCI  p=.52  MCI vs HC  p<.01 |
| Pick, Mellers, & Goldstein, 2016 | WASI Vocabulary* | 51.6 (11.1)* | 55.2 (9.8)* | t(81)=1.6, p=.12 |
|  | WASI Matrix Reasoning* | 54 (10)* | 56 (15)* | U(83)=746, p=.29 |
| *T scores | WASI Full Scale IQ | 103.6 (14.5) | 108.1 (13.1) | t(81)=1.5, p=.14 |
|  | BFRT (all participants) Median (IQR) | 47 (7) n=39 | 49 (5) n=43 | U(82)=635, p=.056 |
|  | BFRT (minus scores <40) Median (IQR) | 49 (7) n=35 | 49 (5) n=42 | U(77)=598.5, p=.16 |
|  | WMS-III Faces I Scaled scores | 10.9 (3.2) n=39 | 11.1 (2.9) n=43 | t(80)=.285, p=.78 |
| Pick, Mellers, & Goldstein, 2018a | WASI Full Scale IQ | 103.8 (14.6) | 108.2 (13.3) | t(79)=1.4, p=.165 |
|  | WASI Vocabulary* | 51.7 (11.2) | 55.4 (9.9) | t(79)=1.5, p=.125 |
| *T scores | WASI Matrix Reasoning* | 54 (10) | 55.5 (15) | U(81)=725.5, p=.376 |
|  | VOSP OD (raw)  Median (IQR) | 18 (3) n=38 | 17 (3) n=42 | U(80)=551.5, p=.016 |
|  | WMS-III Family Pictures Scaled scores | 8.7 (2.4) n=38 | 7.3 (1.9) n=42 | t(78)=−2.99, p=.004 |
| Pick, Mellers, & Goldstein, 2018b | WASI Full Scale IQ | 104.1 (14.7) | 108.1 (13.1) | t(79)=1.29, p=.198 |
|  | WASI Vocabulary* | 51.8 (11.3) | 55.2 (9.8) | t(79)=1.46, p=.148 |
| *T scores | WASI Matrix Reasoning*  Median (IQR) | 54.5 (10) | 56 (15) | U(81)=730, p=0.410 |
|  | BFRT  Median (IQR) | 49 (7) | 49 (5) | U(80)=631, p=0.109 |
|  | Stroop test* | 52.8 (8.3) | 51.5 (9.01) | t(78)=−0.656, p=0.514 |
| Pick et al., 2023 | WASI Full Scale IQ-2 | 104.6 (10.7) | 106.6 (9.2) | t(31)=0.58, p=0.57, g=0.20 |
|  | WASI Vocabulary* | 54.1 (5.7) | 55.0 (8.6) | t(31)=0.34, p=0.73, g=0.12 |
| *T scores | WASI Matrix Reasoning* | 51.4 (8.6) | 52.8 (4.9) | t(24)=0.56, p=0.58, g=0.19 |
|  | Motor Screening Test: Mean Motor Latency | 862.2 (189.7) | 751.8 (199.7) | t(31)=-1.63, p=0.11, g=0.55 |
|  | Reaction Time Test: Mean Reaction Time | 382.2 (41.5) | 361.1 (32.7) | t(31)=-1.62, p=0.12, g=0.55 |
|  | Reaction Time Test: Mean Movement Time | 252.2 (54.6) | 217.9 (55.0) | W=92.0, p=0.12, r=0.27 |
|  | Rapid Visual Information Processing: Median Response Latency | 403.5 (40.1) | 440.0 (85.4) (n=16) | W=179.5, p=0.05, r=0.3 |
|  | Rapid Visual Information Processing: RVIP Ability | 0.89 (0.05) | 0.91 (0.05) (n=16) | t(30)=0.81, p=0.43, g=0.28 |
|  | Rapid Visual Information Processing: Total Misses | 22.4 (10.3) | 21.0 (12.2) | t(31)=-0.37, p=0.72, g=0.12 |
|  | Rapid Visual Information Processing: Probability of Hit | 0.58 (0.19) | 0.61 (0.23) | t(31)=0.37, p=0.72, g=0.12 |
|  | Rapid Visual Information Processing: Probability of False Alarm | 0.006 (0.008) | 0.004 (0.003) (n=16) | W=96.0, p=0.23, r=0.21 |
|  | Spatial Span: Forward Span Length | 7.0 (1.0) | 7.0 (3.0) | W=156.5, p=0.46, r=0.13 |
|  | Spatial Span: Forward Errors | 15.9 (5.1) | 17.2 (8.8) | t(26.0)=0.55, p=0.59, g=0.18 |
|  | Spatial Span: Reverse Span Length | 6.0 (1.5) (n=15) | 6.0 (1.0) | W=107.0, p=0.44, r=0.13 |
|  | Spatial Span: Reverse Errors | 14.4 (3.5) (n=15) | 12.2 (5.7) | t(27.0)=-1.34, p=0.19, g=0.45 |
|  | Intra-Extra Dimensional Set Shift: Total Errors | 14.0 (7.8) (n=12) | 12.0 (8.5) (n=10) | W=55.0, p=0.77, r=0.05 |
|  | Intra-Extra Dimensional Set Shift: Adjusted Errors | 16.0 (28.5) | 22.0 (47.0) | W=152.0, p=0.58, r=0.10 |
|  | Intra-Extra Dimensional Set Shift: Total Trials Completed | 80.7 (16.6) (n=12) | 75.4 (8.3) (n=10) | t(20)=-0.26, p=0.80, g=0.11 |
|  | Intra-Extra Dimensional Set Shift: Completed Stage Trials | 70.0 (25.0) | 71.2 (15.7) | t(31)=0.17, p=0.87, g=0.06 |
|  | Intra-Extra Dimensional Set Shift: Completed Stage Errors | 12.0 (7.75) | 12.0 (11.0) | W=146.5, p=0.72, r=0.06 |
|  | Intra-Extra Dimensional Set Shift: Stages Completed | 9.0 (0.5) | 9.0 (2.0) | W=112.5, p=0.33, r=0.17 |
|  | Intra-Extra Dimensional Set Shift: Response Latency (ms) | 112341 (42752) (n=12) | 125164 (44040) (n=10) | W=73.0, p=0.42, r=0.14 |
|  | Stop Signal Task: Errors Go Trials | 0.0 (1.0) | 0.0 (2.0) | W=138.0, p=0.95, r=0.01 |
|  | Stop Signal Task: Errors Stop Trials | 38.5 (8.5) | 39.0 (6.0) | W=154.5, p=0.52, r=0.11 |
|  | Stop Signal Task: Number of Missed Trials | 4.0 (5.3) | 2.0 (5.0) | W = 105.5, p=.28, r=.19 |
|  | Stop Signal Task: Reaction Time (ms) | 241 (41.2) | 249 (54.2) | t(31)=.44, p=.66, g=.16 |
|  | Emotional Bias Task–Anger: Bias Point | 8.56 (1.4) | 8.82 (1.1) | t(31)=0.60, p=0.55, g=0.21 |
|  | Emotional Bias Task-Anger: Anger (ms) | 772.4 (161.1) | 958.4 (255.1) |  |
|  | Emotional Bias Task-Anger: Happiness (ms) | 726.3 (122.7) (n=15) | 885.1 (198.6) (n=17) |  |
|  | Emotional Bias Task-Disgust: Bias Point | 8.16 (0.86) | 7.94 (1.16) | t(30)=-0.59, p=0.56, g=0.20 |
|  | Emotional Bias Task-Disgust: Disgust (ms) | 684.3 (95.1) | 789.3 (170.0) |  |
|  | Emotional Bias Task-Disgust: Happiness (ms) | 723.4 (97.6) (n=14) | 789.4 (168.5) (n=16) |  |
|  | Emotion Recognition Test: Total Hits | 59.5 (8.25) | 59.5 (7.0) (n=16) | W=145.5, p=0.52, r=0.11 |
|  | Emotion Recognition Test: Total Hits Anger | 8.1 (2.2) | 7.3 (2.4) |  |
|  | Emotion Recognition Test: Total Hits Disgust | 9.8 (3.2) | 10.8 (3.3) |  |
|  | Emotion Recognition Test: Total Hits Fear | 7.2 (2.4) | 7.4 (3.2) |  |
|  | Emotion Recognition Test: Total Hits Happiness | 12.0 (2.2) | 12.4 (1.6) |  |
|  | Emotion Recognition Test: Total Hits Sadness | 11.4 (2.8) | 9.6 (4.1) |  |
|  | Emotion Recognition Test: Total Hits Surprise | 11.1 (1.8) | 11.9 (1.7) (n=16) |  |
|  | Emotion Recognition Test: Unbiased Hit Rate Anger | 0.42 (0.17) | 0.41 (0.17) |  |
|  | Emotion Recognition Test: Unbiased Hit Rate Disgust | 0.42 (0.19) | 0.50 (0.22) |  |
|  | Emotion Recognition Test: Unbiased Hit Rate Fear | 0.32 (0.15) | 0.31 (0.22) |  |
|  | Emotion Recognition Test: Unbiased Hit Rate Happiness | 0.59 (0.13) | 0.66 (0.11) |  |
|  | Emotion Recognition Test: Unbiased Hit Rate Sadness | 0.54 (0.14) | 0.45 (0.20) |  |
|  | Emotion Recognition Test: Unbiased Hit Rate Surprise | 0.50 (0.09) | 0.50 (0.12) (n=16) |  |
|  | Emotion Recognition Test: False Alarms Anger | 2.8 (2.5) | 2.1 (3.0) |  |
|  | Emotion Recognition Test: False Alarms Disgust | 7.4 (6.3) | 6.3 (4.8) |  |
|  | Emotion Recognition Test: False Alarms Fear | 4.5 (3.8) | 6.3 (4.6) |  |
|  | Emotion Recognition Test: False Alarms Happiness | 5.3 (5.2) | 3.4 (2.8) |  |
|  | Emotion Recognition Test: False Alarms Sadness | 5.1 (3.5) | 4.4 (3.4) |  |
|  | Emotion Recognition Test: False Alarms Surprise | 5.4 (2.6) | 8.3 (5.5) (n=16) |  |
|  | Emotion Recognition Test: Median reaction time correct responses | 1039.0 (227.0) | 1048.5 (188.0) (n=16) |  |
|  | Emotion Recognition Test: Median reaction time correct Anger | 1152.5 (521.0) | 1201.6 (405.2) |  |
|  | Emotion Recognition Test: Median reaction time correct Disgust | 1301.2 (470.1) | 1175.9 (223.9) |  |
|  | Emotion Recognition Test: Median reaction time correct Fear | 1362.7 (362.9) | 1660.9 (1155.2) |  |
|  | Emotion Recognition Test: Median reaction time correct Happiness | 926.0 (289.3) | 873.4 (172.1) |  |
|  | Emotion Recognition Test: Median reaction time correct Sadness | 1142.8 (237.8) | 1082.6 (237.7) |  |
|  | Emotion Recognition Test: Median reaction time correct Surprise | 948.1 (272.1) (n=15) | 1050.5 (463.1) (n=16) |  |
| Prigatano & Kirlin, 2009 | RAVLT Delayed Recall T score* | 40.65 (15.49) | 34.00 (15.74) | F=2.04, p=.16 |
| *T score | BVMT-R Delayed Recall T score* | 41.61 (15.85) | 34.91 (15.20) | F=2.09, p=.16 |
|  | BNIS Memory subscale raw score | 4.68 (1.84) | 3.27 (2.14) | F=5.49, p=.02 |
|  | Trail Making Task-B T score* | 42.25 (10.61) | 41.68 (10.29) | F=.03, p=.86 |
|  | BNT T score* | 40.52 (17.33) | 27.77 (19.49) | F=5.39, p=.03 |
|  | BNIS Affect subscale raw score | 2.13 (1.14) | 2.91 (1.02) | F=5.81, p=.02 |
|  | WAIS-III Digit Span age-scaled score | 8.17 (3.17) | 8.00 (2.25) | F=.05, p=.83 |
|  | WASI Verbal IQ standard score | 95.43 (12.03) | 86.95 (13.76) | F=4.86, p=.03 |
|  | WASI Performance IQ standard score | 98.09 (12.25) | 96.23 (16.01) | F=.19, p=.66 |
| Sackellares & Sackellares, 2001 | WAIS-R Full-scale IQ <90 | 16 (40.0%) |  |  |
|  | Halstead impairment index >/= 0.5 | 25 (62.5%) |  |  |
|  | Finger oscillation dominant hand | 38.52 (6.86) | 53.57 (6.81) | p<.0001 |
|  | Finger oscillation nondominant hand | 37.34 (4.82) | 47.98 (5.73) | p<.0001 |
|  | Asymmetry Index-Finger Oscillation | 1.13 (15.69) | 10.12 (9.04) | p=.0013 |
| Salinsky et al., 2020 | RBANS Total Scale Index | Valid TOMM  88.9 (11.2) n=53  Invalid TOMM  70.8 (10.9) n=16 | Valid TOMM  82.3 (12.4) n=55  Invalid TOMM  68.4 (11.6) n=7 | FS valid vs invalid TOMM: p<.001, d=1.36  ES valid vs invalid TOMM: p=.02, d=.92 |
| Schwilk et al., 2021 | Mehrfachwahl-Wortschatztest | 31.9 (3.2) |  |  |
|  | VLMT delayed recall (n=28) | 13.0 (2.0) |  |  |
| *According to age norm 51-60 years | AVLT (n=24) | 13.2 (1.9)  78 (24)* |  |  |
|  | “Zahlenverbindungstest” (n=21) | 81.5 (17.8)  83.7 (14.4)* |  |  |
| Silveri et al., 2022 | Mini-Mental State Examination | 27.4 (2.0) |  |  |
|  | Raven’s coloured progressive matrices | 26.6 (4.0) |  |  |
|  | Reading the Mind in the Eyes Test | 21.83 (5.77) | HC1: 25.71 (2.16) | p=.020 |
| Simani et al., 2020a | WAIS Full Scale IQ | 81.20 (14.37) | GGE: 88.16 (12.76)  HC: 101.31 (17.65) | HCs vs GGE  p < 0.01  HCs vs FS  p < 0.01 |
|  | WAIS Verbal IQ | 82.67 (16.53) | GGE: 90.83 (12.59)  HC: 102.84 (16.50) | HCs vs GGE  p < 0.01  HCs vs FS  p < 0.01 |
|  | WAIS Performance IQ | 80.53 (13.21) | GGE: 85.53 (12.39)  HC: 98.59 (17.46) | HCs vs GGE  p < 0.01  HCs vs FS p < 0.01 |
|  | Full Scale Attention Quotient | 47.83 (32.68) | GGE: 71.83 (35.46)  HC: 98.96 (18.18) | HCs vs. GGE and FS p < 0.001 |
|  | Full Scale Response Control Quotient | 60.18 (35.35) | GGE: 84.83 (36.24)  HC: 104.46 (11.52) | HCs vs GGE p < 0.01  GGE vs FS p < 0.01 |
|  | Auditory attention quotient | 59.63 (31.50) | GGE: 75.76 (34.83)  HC: 99.37 (19.14) | HCs vs. GGE p < 0.01  HCs vs. FS p < 0.01 |
|  | Visual attention quotient | 50.65 (37.04) | GGE: 73.90 (35.09)  HC: 99.28 (14.64) | HCs vs. GGE p < 0.01  HCs vs. FS p < 0.01  GGE vs. FS p < 0.01 |
|  | Auditory vigilance quotient | 50.31 (39.81) | GGE: 71.00 (43.08)  HC: 94.45 (21.17) | HCs vs. GGE p < 0.01  HCs vs. FS p < 0.01 |
|  | Visual vigilance quotient | 49.53 (36.61) | GGE: 77.76 (36.88)  HC: 100.34 (16.82) | HCs vs. GGE p < 0.01  HCs vs. FS p < 0.01  GGE vs. FS p < 0.01 |
|  | Auditory focus quotient | 62.56 (23.30) | GGE: 78.83 (27.89)  HC: 93.71 (11.04) | HCs vs. GGE p < 0.01  HCs vs. FS p < 0.01  GGE vs. FS p < 0.01 |
|  | Visual focus quotient | 63.90 (33.89) | GGE: 83.73 (32.59)  HC: 99.50 (14.08) | HCs vs. GGE p < 0.01  HCs vs. FS p < 0.01 |
|  | Auditory speed quotient | 90.70 (33.56) | GGE: 96.21 (29.49)  HC: 111.18 (16.03) | HCs vs. FS p < 0.01 |
|  | Visual speed quotient | 69.50 (32.06) | GGE: 75.66 (33.18)  HC: 97.12 (11.82 | HCs vs. GGE p < 0.01  HCs vs. FS p < 0.01 |
|  | Auditory response control quotient | 63.08 (30.98) | GGE: 88.10 (26.94)  HC: 98.50 (12.64) | HCs vs. FS p < 0.01  GGE vs. FS p < 0.01 |
|  | Visual response control quotient | 71.36 (35.75) | GGE: 87.93 (38.23)  HC: 109.78 (12.45) | HCs vs. GGE p < 0.01  HCs vs. FS p < 0.01 |
|  | Auditory prudence quotient | 70.58 (32.21) | GGE: 97.00 (28.57)  HC: 99.20 (14.72) | HCs vs. FS p < 0.01  GGE vs. FS p < 0.01 |
|  | Visual prudence quotient | 73.62 (36.76) | GGE: 89.15 (33.74)  HC: 104.37 (9.59) | HCs vs. GGE p < 0.01  HCs vs. FS p < 0.01 |
|  | Auditory consistency quotient | 62.55 (26.50) | GGE: 77.03 (27.97)  HC: 88.84 (17.08) | HCs vs. FS p < 0.01 |
|  | Visual consistency quotient | 72.75 (34.43) | GGE: 87.95 (37.76)  HC: 107.45 (15.57) | HCs vs. FS p < 0.01  GGE vs. FS p < 0.01 |
|  | Auditory stamina quotient | 82.79 (30.63) | GGE: 93.21 (36.74)  HC: 103.10 (15.57) | HCs vs. GGE p < 0.01  HCs vs. FS p < 0.01 |
|  | Visual stamina quotient | 82.31 (36.66) | GGE: 82.61 (39.42)  HC: 107.71 (14.52) | HCs vs. GGE p < 0.01  HCs vs. FS p < 0.01 |
| Simani et al., 2020b | WAIS Full Scale IQ | 83.30 (12.35) | IGE: 88.76 (11.91)  HC: 88.82 (10.23) | p=.176 |
|  | IVA-CPT Full Scale Attention Quotient | 46.76 (32.11) | IGE: 71.83 (35.46)  HC: 98.96 (18.18) | HC vs FS p=.001  HC vs IGE p=.001  IGE vs FS p=.017 |
|  | IVA-CPT Full Scale Response Control Quotient | 59.10 (34.52) | IGE: 84.83 (36.42)  HC: 104.46 (11.52) | HC vs FS p=.001  HC vs IGE p=.02  IGE vs FS p=.02 |
| Slater et al., 1995 | WAIS-R Block Design | 8.375 (2.841) | 7.767 (2.239) | p=.383 |
|  | Judgment of Line Orientation | 6.714 (2.077) | 7.0 (2.407) | p=.661 |
|  | WAIS-R Similarities | 8.417 (2.733) | 8.0 (2.435) | p=.557 |
|  | WAIS-R Proverb Interpretation  Multiple Choice | 16.150 (3.856) | 16.370 (4.305) | p=.857 |
|  | WAIS-R Proverb Interpretation  Spontaneous | 5.794 (1.480) | 5.920 (2.090) | p=.832 |
|  | WAIS-R Proverb Interpretation  Information | 7.957 (2.738) | 7.714 (2.386) | p=.741 |
|  | WAIS-R Proverb Interpretation Arithmetic | 8.417 (2.918) | 8.655 (3.210) | p=.78 |
|  | Boston Naming Test | 48.636 (8.483) | 44.690 (8.897) | p=.116 |
|  | Controlled Word Association Test | 31.292 (10.630) | 30.967 (11.183) | p=.914 |
|  | WAIS-R Digit Span | 7.917 (2.483) | 9.276 (2.737) | p=.066 |
|  | WMS Logical Memory, Immediate | 12.083 (3.611) | 10.4 (3.519) | p=.09 |
|  | WMS Logical Memory, Delayed | 10.708 (3.210) | 8.333 (3.818) | p=.018 |
|  | Paired Associations, Easy | 5.9 (0.308) | 5.760 (0.523) | p=.296 |
|  | Paired Associations, Hard | 2.950 (1.356) | 2.520 (1.447) | p=.314 |
|  | Benton Visual Retention Test | 12.250 (2.266) | 12.783 (3.450) | p=.592 |
| Strutt et al., 2011  *Standard score rather than scale score  ^Scale score | Sattler’s Equation (IQ)* | 90.2 (9.63) | 96.4 (9.32) | F=5.38, p=.03 |
|  | WAIS-III Digit Span^ | 7.61 (2.36) | 9.12 (2.42) | F=5.51, p=.02 |
|  | WAIS-III Arithmetic^ | 7.28 (2.00) | 8.80 (2.29) | F=2.08, p=.16 |
|  | WAIS-III Letter-Number Sequencing^ | 7.50 (2.08) | 7.92 (1.68) | F=.02, p=.97 |
|  | WAIS-III Working Memory Index* | 83.6 (12.2) | 89.4 (10.0) | F=1.17, p=.064 |
|  | Spatial Span^ | 6.45 (2.42) | 8.77 (2.41) | F=11.8, p=.001 |
|  | Trail Making Task-A ^ | 7.13 (4.37) | 8.70 (3.47) | F=1.83, p=.18 |
|  | Mental Control^ | 7.26 (2.79) | 8.09 (1.92) | F=.78, p=.40 |
|  | Logical Memory I^ | 8.00 (2.90) | 6.05 (1.53) | F=8.33, p=.006 |
|  | Logical Memory II^ | 8.81 (2.97) | 6.00 (1.77) | F=15.6, p<.001 |
|  | Verbal Paired Associates I^ | 7.87 (2.25) | 5.50 (2.06) | F=15.3, p<.001 |
|  |  |  |  |  |
|  | Verbal Paired Associates II^ | 8.48 (2.28) | 6.05 (2.61) | F=13.1, p=.001 |
|  | Visual Reproduction I^ | 7.47 (3.60) | 9.95 (3.17) | F=6.70, p=.01 |
|  | Visual Reproduction II^ | 8.70 (2.73) | 10.70 (3.20) | F=5.78, p=.02 |
|  | Boston Naming Test^ | 6.36 (4.07) | 4.52 (3.74) | F=.70, p=.41 |
|  | Letter Fluency^ | 6.87 (3.29) | 7.61 (2.86) | F=.02, p=.89 |
|  | Semantic Fluency^ | 7.35 (2.92) | 8.69 (1.97) | F=1.91, p=.17 |
|  | Trail Making Task-B^ | 7.30 (3.95) | 9.45 (3.10) | F=4.51, p=.04 |
|  | Similarities^ | 8.76 (2.45) | 9.09 (2.07) | F=.25, p=.62 |
| Tyson et al., 2018  SS: standard score (M = 100, SD = 15)  *ACSS: Age-corrected scaled score (M = 10, SD = 3) | WAIS Full Scale IQ (SS) | 95.7 (16.9) | 84.4 (18.4) | p<.01, d=.64 |
|  | Vocabulary* | 10.3 (3.6) | 7.6 (3.6) | p<.01, d=.73 |
|  | Similarities* | 9.4 (9.4) | 7.7 (3.3) | p=.03, d=.47 |
|  | Information* | 9.9 (3.6) | 7.2 (3.0) | p<.01, d=.81 |
|  | Block Design* | 9.3 (2.8) | 8.1 (3.6) | p=.10 |
|  | Matrix Reasoning* | 9.8 (3.3) | 8.2 (3.4) | p=.03, d=.49 |
|  | Digit Span* | 8.8 (3.0) | 7.4 (3.3) | p=.05, d=.45 |
|  | Vocabulary-DS* | 1.7 (3.4) | 0.2 (3.2) | p=.04, d=.45 |
|  | Arithmetic* | 9.5 (3.8) | 7.0 (3.7) | p<.01, d=.66 |
|  | Symbol Search* | 8.5 (3.5) | 6.8 (3.8) | p=.04, d=.46 |
|  | Coding* | 7.6 (3.1) | 6.9 (2.8) | p=.26 |
|  | WRAT Reading (SS) | 97.3 (15.5) | 85.9 (18.7) | p=.006, d=.66 |
|  | Boston Naming Test (T-score) | 44.8 (10.9) | 34.6 (10.5) | p<.001, d=.95 |
|  | Complex Ideational Material (T-score) | 42.2 (13.7) | 37.1 (15.0) | p=.11 |
|  | Logical Memory I* | 8.7 (3.3) | 7.5 (3.8) | p=.200 |
|  | Logical Memory II* | 9.0 (3.9) | 7.3 (3.8) | p=.04, d=.45 |
|  | Logical Memory Recognition (Raw) | 25.0 (3.4) | 23.5 (3.5) | p=.04, d=.46 |
|  | CVLT-II Trials 1-5 (T-score) | 41.4 (11.4) | 39.9 (13.4) | p=.57 |
|  | CVLT-II SD-FR (z-score) | -1.15 (1.4) | -1.17 (1.5) | p=.95 |
|  | CVLT-II LD-FR (z-score) | -1.23 (1.4) | -1.48 (1.6) | p=.44 |
|  | CVLT-II d’ (z-score) | -0.85 (1.46) | -0.89 (1.71) | p=.89 |
|  | CVLT-II FCR (Raw) | 14.7 (2.4) | 15.8 (0.7) | p=.002, d=.57 |
|  | Controlled Word Association (T-score) | 42.1 (11.9) | 32.0 (12.4) | p=.001, d=.83 |
|  | Animals (T-score) | 36.2 (15.8) | 32.8 (14.1) | p=.29 |
|  | WCST TE (T-score) | 46.1 (10.6) | 42.6 (13.0) | p=.21 |
|  | WCST PE (T-score) | 47.5 (13.0) | 43.4 (15.2) | p=.22 |
|  | WCST FMS (Raw) | 1.1 (1.0) | 0.7 (1.2) | p=.13 |
|  | Finger tapping test (T-score) | 42.0 (16.7) | 37.0 (10.5) | p=.11 |
|  | D-KEFS Trails 1* | 8.8 (3.7) | 8.8 (3.8) | p=.99 |
|  | D-KEFS Trails 2* | 8.9 (3.8) | 7.5 (3.9) | p=.15 |
|  | D-KEFS Trails 3* | 8.3 (3.5) | 7.2 (4.1) | p=.29 |
|  | D-KEFS Trails 4* | 8.8 (3.8) | 7.5 (3.8) | p=.21 |
|  | D-KEFS Trails 5* | 10.0 (3.2) | 9.8 (3.7) | p=.75 |
| Vechetova et al., 2022 | Mini-Mental State Examination | 27.2 (2) | 28.3 (1) | p=.010 |
|  | AVLT sum | 43.89 (8.6) | 49.10 (8.9) | p=.029, d=.59 |
|  | AVLT B | 4.19 (1.4) | 5.73 (1.8) | p<.001, d=.97 |
|  | AVLT free recall | 9.93 (2.9) | 10.10 (2.6) | p=.81, d=.06 |
|  | AVLT delay recall | 9.41 (3.0) | 9.97 (2.9) | p=.47, d=.19 |
|  | AVLT recognition hits | 13.89 (1.4) | 13.47 (1.4) | p=.25, d=.31 |
|  | 15-item Boston Naming Test | 13.78 (1.2) | 14.33 (1.0) | p=.056, d=.51 |
|  | Digit span | 13.93 (2.3) | 15.53 (2.9) | p=.026, d=.60 |
|  | WAIS-III Digit span forwards | 8.22 (1.3) | 8.97 (1.7) | p=.077, d=.48 |
|  | WAIS-III Digit span backwards | 6.22 (3.1) | 6.57 (1.6) | p=.59, d=.15 |
|  | Digit symbols | 56.63 (16.3) | 67.57 (15.2) | p=.011, d=.70 |
|  | N-back ratio | 0.62 (0.2) | 0.73 (0.2) | p=.051, d=.53 |
|  | ROCF copy | 32.07 (2.7) | 31.80 (3.1) | p=.73, d=.09 |
|  | ROCF 3 | 20.54 (5.5) | 21.22 (4.7) | p=.62, d=.13 |
|  | ROCF 30 | 20.22 (5.2) | 20.80 (4.6) | p=.66, d=.12 |
|  | Stroop test W | 79.22 (15.2) | 88.43 (15.1) | p=.026, d=.61 |
|  | Stroop test C | 63.48 (15.1) | 72.87 (10.6) | p=.008, d=.73 |
|  | Stroop test CW | 35.37 (12.2) | 43.13 (8.5) | p=.007, d=.74 |
|  | Stroop test IF | 0.75 (8.3) | 3.28 (5.9) | p=.20, d=.35 |
|  | Trail Making Task-A (s) | 36.15 (15.1) | 27.87 (8.6) | p=.013, d=.68 |
|  | Trail Making Task-B (s) | 85.78 (38.1) | 75.07 (41.8) | p=.32, d=.27 |
|  | Verbal fluency animals | 25.07 (5.6) | 28.33 (6.1) | p=.041, d=.55 |
|  | Verbal fluency KPS | 42.89 (9.9) | 53.73 (8.6) | p<.001, d=1.17 |
|  | Verbal fluency vegetables | 14.37 (4.1) | 16.23 (3.5) | p=.071, d=.49 |
|  | Domain: Attention | -.72 (.9) | 0 (.8) | p=.005 |
|  | Domain: Memory | -.26 (.9) | 0 (.8) | p>.99 |
|  | Domain: Executive functions | -.63 (.8) | 0 (.6) | p=.01 |
|  | Domain: Speech and language | -.54 (.7) | 0 (.7) | p=.025 |
|  | Domain: Visuospatial functions | -.03 (.8) | 0 (.9) | p>.99 |
|  | Domain: Cognitive performance | -.44 (.6) | 0 (.6) | p=.025 |
| Voon et al., 2013 | WTAR VIQ | 107.24 (9.99) | 108.65 (10.76) | t=-.49, p=.63 |
|  | WTAR PIQ | 106.07 (7.71) | 106.97 (8.71) | t=-.38, p=.71 |
|  | WTAR FSIQ | 107.55 (10.20) | 108.61 (10.86) | t=-.35, p=.73 |
|  | CPT Commission errors | 50.22 (8.61) | 43.15 (7.91) | t=3.31, p=.001 |
|  | CPT Hit reaction time | 49.32 (10.15) | 51.44 (8.32) | t=.88, p=.38 |
|  | CPT Perseveration | 50.44 (13.99) | 47.32 (7.11) | t=1.09, p=.28 |
|  | CPT Omission errors | 49.10 (10.83) | 47.26 (9.16) | t=.71, p=.48 |
|  | CPT Hit RT block change | 48.22 (11.41) | 46.93 (10.23) | t=.46, p=.65 |
|  | CPT Hit SE block change | 53.71 (10.25) | 54.33 (8.94) | t=.25, p=.80 |
|  | WAIS-III Symbol Search | 10.13 (2.08) | 10.69 (3.21) | t=-.17, p=.87 |
|  | WAIS-III Digit Symbol | 10.10 (2.73) | 10.45 (3.56) | t=-.48, p=.94 |
|  | D-KEFS Tower test | 10.15 (4.18) | 9.82 (2.94) | t=.41, p=.69 |
|  | D-KEFS Color-Word Interference  Inhibition | 9.36 (3.57) | 9.64 (3.52) | t=-.11, p=.91 |
|  | Inhibition/Switch | 9.51 (3.86) | 9.97 (3.70) | t=-.48, p=.63 |
|  | Color naming | 8.82 (3.37) | 8.67 (3.32) | t=.07, p=.95 |
|  | Word reading | 8.71 (3.91) | 9.54 (3.28) | t=-.96, p=.34 |
|  | D-KEFS letter fluency | 10.79 (3.13) | 11.31 (4.56) | t=-.59, p=.56 |
|  | D-KEFS category fluency | 10.26 (2.84) | 12.02 (4.45) | t=-2.08, p=.04 |
|  | Boston Naming Test | 45.39 (11.64) | 43.87 (10.64) | t=.58, p=.57 |
|  | BVMT learning | 52.74 (13.49) | 52.50 (14.19) | t=.07, p=.94 |
|  | BVMT recall | 41.75 (13.59) | 45.21 (16.19) | t=-.65, p=.5 |
|  | HVLT retention | 46.37 (10.71) | 45.59 (13.04) | t=.45, p=.66 |
|  | HVLT delayed recall | 47.44 (8.73) | 44.92 (13.55) | t=1.24, p=.22 |
|  | HVLT recognition | 49.71 (9.47) | 48.72 (9.63) | t=.36, p=.72 |
|  | WAIS-III Digit Symbol | 10.10 (2.73) | 10.45 (3.56) | t=-.48, p=.94 |
|  | RBANS JLO, total score | 16.11 (3.24) | 14.68 (3.59) | t=1.56, p=.11 |
|  | RBANS JLO, z score* | 28.20 (18.24) | 28.56 (19.90) | t=.1, p=.92 |
| Walterfang et al., 2012 | NUCOG total | 90.8 (8.0) | ES: 89.6 (8.0)  OS: 91.3 (5.7) | F=.53, p=.663  NUCOG profile between groups:  no effect of group F[3,157] = 0.529, P = 0.663  no group×subscale effect F[3,157] = 1.119, P = 0.292 |
|  | NUCOG attention | 17.5 (2.4) | ES: 17.6 (2.3)  OS: 17.3 (2.2) | F=.23, p=.877 |
|  | NUCOG visuoconstructional | 18.4 (1.7) | ES: 18.7 (1.5)  OS: 19.1 (1.0) | F=1.33, p=.266 |
|  | NUCOG memory | 17.2 (2.9) | ES: 16.7 (2.9)  OS: 17.0 (3.1) | F=.47, p=.707 |
|  | NUCOG executive | 18.2 (2.1) | ES: 17.4 (2.7)  OS: 18.3 (1.7) | F=1.78, p=.153 |
|  | NUCOG language | 19.5 (1.3) | ES: 19.2 (1.1)  OS: 19.5 (0.6) | F=1.02, p=.385 |
| Wilkus & Dodrill 1989 | WAIS Verbal IQ | 99.12 (12.71) | PE: 99.72 (14.21)  GEA: 95.32 (13.15)  GEB: 85.84 (15.69) | FS, PE & GEA F=.80, p=ns  FS & GEB F=3.29 , p<.01 |
|  | WAIS Performance IQ | 95.32 (12.51) | PE: 99.80 (10.86)  GEA: 89.96 (14.52)  GEB: 79.92 (16.71) | FS, PE & GEA F=3.74, p=ns  FS & GEB F=3.69 , p<.001 |
|  | WAIS Full Scale IQ | 97.32 (11.30) | PE: 99.76 (12.68)  GEA: 92.52 (13.3)  GEB: 82.24 (16.36) | FS, PE & GEA F=2.19, p=ns  FS & GEB F=3.79 , p<.001 |
|  | Stroop, Part I | 100.25 (35.77) | PE: 100.25 (25.43)  GEA: 112.96 (55.04)  GEB: 146.04 (62.04) | FS, PE & GEA F=.78, p=ns  FS & GEB F=-3.12, p<.01 |
|  | Stroop, Part II-I | 159.33 (54.84) | PE: 145.42 (43.49)  GEA: 171.79 (68.62)  GEB: 197.75 (79.92) | FS, PE & GEA F=1.30, p=ns  FS & GEB F=-1.93, p=ns |
|  | WMS Logical Memory | 21.04 (6.71) | PE: 17.88 (7.67)  GEA: 19.36 (6.60)  GEB: 15.12 (6.51) | FS, PE & GEA F=1.27, p=ns  FS & GEB F=3.13, p<.01 |
|  | WMS Visual reproduction | 9.40 (2.87) | PE: 9.84 (2.61)  GEA: 8.84 (3.28)  GEB: 7.17 (3.75) | FS, PE & GEA F=.73, p=ns  FS & GEB F=2.35, p=ns |
|  | Perceptual Exam | 17.32 (21.78) | PE: 6.92 (12.78)  GEA: 12.60 (17.13)  GEB: 21.04 (24.61) | FS, PE & GEA F=2.18, p=ns  FS & GEB F=.57, p=ns |
|  | Speech Perception | 9.36 (8.64) | PE: 7.20 (3.42)  GEA: 6.52 (4.49)  GEB: 9.67 (8.22) | FS, PE & GEA F=1.55, p=ns  FS & GEB F=-.13, p=ns |
|  | Name writing, total | 0.74 (0.29) | PE: 0.82 (0.25)  GEA: 0.87 (0.43)  GEB: 0.58 (0.26) | FS, PE & GEA F=.90, p=ns  FS & GEB F=2.05, p=ns |
|  | Category Test | 48.29 (32.06) | PE: 44.56 (27.80)  GEA: 65.76 (32.88)  GEB: 80.56 (29.00) | FS, PE & GEA F=3.33, p=ns  FS & GEB F=-3.70, p<.001 |
|  | Seashore Rhythm | 23.88 (3.97) | PE: 24.80 (3.43)  GEA: 23.60 (4.27)  GEB: 20.72 (5.05) | FS, PE & GEA F=.65, p=ns  FS & GEB F=2.46, p=ns |
|  | Seashore Tonal Memory | 21.08 (6.84) | PE: 21.60 (5.46)  GEA: 17.80 (6.62)  GEB: 14.20 (7.56) | FS, PE & GEA F=2.64, p=ns  FS & GEB F=3.37, p<.01 |
|  | Tapping, total | 83.40 (13.02) | PE: 89.56 (16.14)  GEA: 85.84 (11.86)  GEB: 76.16 (15.69) | FS, PE & GEA F=1.27, p=ns  FS & GEB F=1.78, p=ns |
|  | Trail Making Task-B | 94.12 (52.22) | PE: 68.28 (26.72)  GEA: 116.04 (73.94)  GEB: 178.80 (90.68) | FS, PE & GEA F=4.81, p<.01  FS & GEB F=-4.05, p<.001 |
|  | Aphasia Screening Test | 3.28 (3.27) | PE: 2.68 (2.66)  GEA: 3.00 (2.78)  GEB: 6.96 (7.48) | FS, PE & GEA F=.26, p=ns  FS & GEB F=-2.25, p=ns |
|  | Halstead Imp. Index | 0.53 (0.24) | PE: 0.46 (0.23)  GEA: 0.57 (0.32)  GEB: 0.73 (0.27) | FS, PE & GEA F=1.12, p=ns  FS & GEB F=-2.78, p<.01 |
|  | Scores outside normal limits (%) | 51.16 (23.79) | PE: 47.08 (25.51)  GEA: 57.64 (30.92)  GEB: 75.72 (24.64) | FS, PE & GEA F=.98, p=ns  FS & GEB F=-3.59, p<.001 |
| Wilkus, Dodrill, & Thompson, 1984 | WAIS Verbal IQ | 99.12 | 102.48 | t=.94 |
|  | WAIS Performance IQ | 95.32 | 98.04 | t=.76 |
|  | WAIS Full-Scale IQ | 97.32 | 100.60 | t=.99 |
|  | Neuropsychological Battery: summary – percentage of scores outside normal limits | 51.16 | 45.96 | t=-.82 |
| Ye et al., 2020 | Neuropsychiatry Unit Assessment Tool | 87.0 (9.3) | ES: 86.9 (9.4)  ND: 90.9 (7.4) | Statistics not provided |

Notes. WMS = Wechsler Memory Scale; HLVT = Hopkins Verbal Learning Test; RMT = recognition memory test; SDMT = Symbol Digit Modalities Test; VOSP = Visual Object and Space Perception Battery; WCST = Wisconsin card sorting test; AVLT = Auditory Verbal Learning Test; CVMT = Continuous Visual Memory Test; CVLT = California Verbal Learning Test; WAIS = Wechsler Adult Intellligence Scale; RBMT = Rivermead Behavioural Memory Test; ROCFT = Rey-Osterrieth Complex Figure Test; BADS = Behavioural Assessment of Dysexecutive Syndrome; AVLT = Auditory Verbal Learning Test; BJLOT = Benton Judgment of Line Orientation Test; CT = Cancellation Test; CPT-III = Conner’s Continuous Performance Test; ANT = Attention Network Test; BVMT-R = Brief Visuospatial Memory Test-Revised; WASI = Wechsler Abbreviated Scale of Intelligence; D-KEFS = Delis-Kaplan Executive Function System; WTAR = Wechsler Test of Adult Reading; SWM = Spatial Working Memory; SOC = Stockings of Cambridge; VMPT = Verbal Memory Processes Test; BFRT = Benton Facial Recognition Test; BNIS = BNI screen for higher cerebral functions; RBANS = Repeatable Battery for the Assessment of Neuropsychological Status; IVA-CPT = Integrated Visual and Auditory Continuous Performance Test; WRAT = Wide Range Achievement Test; HVLT = Hopkins Verbal Learning Test; NUCOG = Neuropsychiatry Unit Cognitive Assessment Tool; FS = functional seizures; HC = healthy controls; ES = epilepsy; ND = non-diagnostic; PE = partial epileptic; GEA = generalised epileptic A; GEB = generalised epileptic B; OS = other seizures; FMS = functional motor symptoms; LTLE = left temporal lobe epilepsy; IGE = idiopathic generalised epilepsy; GGE = genetic generalised epilepsy; MCI = mild cognitive impairment; AD = Alzheimer’s disease; FCD = functional cognitive disorder; OMD = organic movement disorder; TLE = temporal lobe epilepsy; GTS = Gilles de la Tourette syndrome; PD = psychiatric diagnoses; SSRD = somatic symptom and related disorders; ET = essential tremor; IPD = idiopathic Parkinson’s disease; LTF = left temporal foci; RTF = right temporal foci; CD = cognitive disorders.
